# The African swine fever virus MGF300-4L protein is associated with viral pathogenicity by promoting the autophagic degradation of IKK*β* and increasing the stability of I*κ*B*α*

**DOI:** 10.1080/22221751.2024.2333381

**Published:** 2024-03-19

**Authors:** Tao Wang, Rui Luo, Jing Zhang, Jing Lan, Zhanhao Lu, Huanjie Zhai, Lian-Feng Li, Yuan Sun, Hua-Ji Qiu

**Affiliations:** a State Key Laboratory for Animal Disease Control and Prevention, National African Swine Fever Para-Reference Laboratory, National High Containment Facilities for Animal Diseases Control and Prevention, Harbin Veterinary Research Institute, Chinese Academy of Agricultural Sciences, Harbin, People’s Republic of China; b College of Animal Sciences, Yangtze University, Jingzhou, People’s Republic of China

**Keywords:** African swine fever virus, MGF300-4L, IKK*β*, I*κ*B*α*, chaperone-mediated autophagy

## Abstract

African swine fever (ASF) is a highly contagious, often fatal viral disease caused by African swine fever virus (ASFV), which imposes a substantial economic burden on the global pig industry. When screening for the virus replication-regulating genes in the left variable region of the ASFV genome, we observed a notable reduction in ASFV replication following the deletion of the *MGF300-4L* gene. However, the role of MGF300-4L in ASFV infection remains unexplored. In this study, we found that MGF300-4L could effectively inhibit the production of proinflammatory cytokines IL-1*β* and TNF-*α*, which are regulated by the NF-*κ*B signaling pathway. Mechanistically, we demonstrated that MGF300-4L interacts with IKK*β* and promotes its lysosomal degradation via the chaperone-mediated autophagy. Meanwhile, the interaction between MGF300-4L and I*κ*B*α* competitively inhibits the binding of the E3 ligase *β*-TrCP to I*κ*B*α*, thereby inhibiting the ubiquitination-dependent degradation of I*κ*B*α*. Remarkably, although ASFV encodes other inhibitors of NF-*κ*B, the *MGF300-4L* gene-deleted ASFV (Del4L) showed reduced virulence in pigs, indicating that MGF300-4L plays a critical role in ASFV pathogenicity. Importantly, the attenuation of Del4L was associated with a significant increase in the production of IL-1*β* and TNF-*α* early in the infection of pigs. Our findings provide insights into the functions of MGF300-4L in ASFV pathogenicity, suggesting that MGF300-4L could be a promising target for developing novel strategies and live attenuated vaccines against ASF.

## Introduction

African swine fever virus (ASFV) is the causative agent of African swine fever (ASF), which is a highly contagious disease affecting domestic pigs and wild boar with a mortality of up to 100%. ASF remains endemic in Sub-Saharan African countries and spread to the Caucasus region of Europe in 2007 [1]. Subsequently, the disease quickly spread to Russia, several member countries of the European Union, China, and numerous Southeast Asian countries, leading to substantial economic losses to the pig industry [2,3]. Presently, the unavailability of desirable ASF vaccines is primarily due to an inadequate understanding of the underlying mechanism of ASFV pathogenicity [4,5].

ASFV is a large double-stranded DNA virus that belongs to the *Asfarviridae* family. The ASFV genome consists of a conserved central region of approximately 100 kilobase pairs (kb), as well as a left variable region (LVR) of about 45 kb and a right variable region (RVR) of about 25 kb [6]. The LVR and RVR regions encompass five multigene family genes (MGFs), namely MGF100, MGF110, MGF300, MGF505, and MGF360 [7]. Although the proteins encoded by *MGFs* are not essential for viral replication *in vitro*, they play important roles in virus pathogenicity, host range, and immune regulation [7–10]. For example, the MGF360-9L protein has been demonstrated to degrade STAT1 and STAT2, inhibiting the secretion of interferon beta (IFN-*β*) and associated with the virulence of ASFV [11,12]. Additionally, the MGF505-7R protein of ASFV suppresses type I IFN production by interacting with the key molecules in the cGAS-STING pathway, including IRF3, IRF7, IRF9, TBK1, IKK*α*, and STING [12,13]. However, the functions of the proteins encoded by *MGFs* and their roles in ASFV pathogenicity require further elucidation [14].

The mammalian antiviral immune responses induced by type I IFNs and proinflammatory cytokines, including IL-1*β* and TNF-*α*, represent the first line of host defense upon infection [15,16]. The transcription factor NF-*κ*B acts as a central signal regulator in inducing both IFNs and proinflammatory responses [17]. The activation of the NF-*κ*B occurs through two distinct pathways: the canonical pathway (CP) and the alternative pathway (AP) [18]. CP is initiated by a cascade of events that begin with the activation of various pattern recognition receptors (PRRs) and cytokine receptors. Activated PRRs trigger the activation of the NF-*κ*B kinase (IKK) complex, which consists of IKK*α*, IKK*β*, and NEMO [19]. Upon IKK being activated, IKK*β* phosphorylates I*κ*B*α* on the serines at positions 32 and 36, triggering the recognition and ubiquitination by beta-transducin repeat-containing protein (*β*-TrCP). Subsequently, the ubiquitinated I*κ*B*α* undergoes degradation by the 26S proteasome [20]. As a result, the NF-*κ*B complexes are released from their inhibitor (I*κ*B*α*) and translocated into the nucleus, initiating the expression of various downstream genes, including IFNs, chemokines, costimulatory molecules, and proinflammatory cytokines [21]. In response to this challenge, viruses have evolved diverse strategies to counteract the host antiviral immune response mediated by NF-*κ*B to ensure their survival. For instance, the cytoplasmic DNA virus vaccinia virus (VACV) encodes multiple proteins that suppress the activation of the NF-*κ*B signaling pathway at various levels [22,23]. Several studies show that ASFV encodes various proteins, such as MGF300-2R, A238L, H240R, MGF505-7R, and F317L, to inhibit the NF-*κ*B signaling pathway and evade the host innate immunity [24–26]. Among them, recent studies have highlighted the roles of MGF300-2R, H240R, and MGF505-7R in viral pathogenicity, underscoring the significance of inhibiting the NF-*κ*B-mediated host antiviral response during ASFV infection [25–27]. Understanding the functions of *MGFs*-encoded proteins and their association with ASFV pathogenicity has become a current hotspot in the field.

Autophagy is a powerful mechanism employed by the host to defend against viral infections [28]. Macroautophagy, chaperone-mediated autophagy (CMA), and microautophagy are the three types of autophagy present in most mammalian cells. Macroautophagy, the most well-studied autophagy pathway, begins with the formation of the autophagosome's limiting membrane [29]. In contrast, CMA is a selective degradation process that specifically targets proteins containing a pentapeptide CMA targeting motif, also known as a KFERQ motif, for lysosomal degradation [30]. CMA facilitates the lysosomal degradation of proteins by recognizing a pentapeptide motif in the protein sequence by the chaperone heat shock cognate 71-kDa protein (HSC70). Following this, the substrate/HSC70 complex binds to the essential receptor for CMA, the lysosome-associated membrane protein type 2A (LAMP2A) [30], which promotes the multimerization of LAMP2A into a complex. Subsequently, the substrate proteins are translocated into the lysosome for degradation. The proteins encoded by ASFV, such as A137R, L83L, and MGF300-2R, can exploit the macroautophagy pathway to promote virus infection and pathogenicity [31]. However, the role of CMA in ASFV infection remains unclear.

The MGF300 proteins (1L, 2R, and 4L) are located in the LVR of the ASFV genome, with amino acid identities ranging from 25% to 46% [32]. However, the functions of the MGF300 proteins have remained elusive since their discovery. Previously, we have discovered that the MGF300 proteins may be involved in adapting ASFV to human embryonic kidney cells (HEK293T) cells during the early stage of viral infection [33]. Furthermore, we have demonstrated that the cell-adapted ASFV, which has a 24.5-kb gene-deletion in the LVR, loses its ability to replicate in primary porcine alveolar macrophages (PAMs) [34]. Additionally, by deleting different sets of *MGFs* in the LVR from the highly virulent ASFV HLJ/18 strain, we have revealed the critical role of MGF300 in viral replication in PAMs [26]. In further analysis, we have identified MGF300-2R as a new virulence factor critical to viral replication both *in vitro* and *in vivo*. Remarkably, we have shown that MGF300-2R promotes the K27-linked ubiquitination of IKK*α* and IKK*β*, leading to the TOLLIP-dependent autophagic degradation [26]. Nevertheless, the exact mechanisms underlying the regulation of ASFV replication in PAMs by MGF300-1L and MGF300-4L remain largely unexplored.

In the present study, we demonstrated that MGF300-4L functions as a virulence factor that can inhibit the production of IL-1*β* and TNF-*α* both *in vitro* and *in vivo*. Mechanistically, MGF300-4L interacts with both IKK*β* and I*κ*B*α*, resulting in the inhibition of p65 nuclear translocation and the production of the IL-1*β* and TNF-*α*. Collectively, our findings elucidate dual-target mechanisms of MGF300-4L as a virulence determinant involved in modulating inflammation and provide novel insights for the development of live attenuated vaccines against ASF.

## Materials and methods

### Ethics statements

All the experiments involving live ASFV manipulation in this study were performed in the biosafety level 3 facilities (BSL3) at Harbin Veterinary Research Institute (HVRI) of the Chinese Academy of Agricultural Sciences (CAAS) and approved by the Ministry of Agriculture and Rural Affairs, China. This study was conducted strictly with the Animal Welfare Act and the Guide for the Care and Use of Laboratory Animals, approved by the Laboratory Animal Welfare Committee of HVRI, with the approval number 230724-01-GR.

### Cells and viruses

HEK293T cells were cultured in Dulbecco’s Modified Eagle’s medium (DMEM, Gibco, catalog no. C11995500BT), supplemented with 10% fetal bovine serum (FBS, Gibco, catalog no. A5669701). *ATG5*-knockout (KO) HeLa cells, *ATG3*-KO, *ATG7*-KO, *TOLLIP*-KO, and *p62*-KO HEK293T cells were previously reported [26,35]. The HeLa cell line (catalog no. C8001) stably expressing p65 fused with the enhanced green fluorescent protein (EGFP) was purchased from Beyotime. PAMs were isolated from the lung lavage fluid of 28-day-old healthy specific-pathogen-free (SPF) piglets and maintained in RPMI-1640 medium with L-glutamine (Thermo Fisher Scientific, catalog no. C11875500BT) supplemented with 10% (v/v) FBS and antibiotics (100 U/mL of penicillin and 100 mg/mL of streptomycin). All the cells were maintained in an incubator with 5% CO_2_ at 37°C. The ASFV HLJ/18 strain [36] was used to construct the *MGF300-4L*-deleted ASFV (Del4L) and for the experiments involving the wild-type ASFV (ASFV-WT). The cell-adapted ASFV (ASFV-P121) and Del4L were generated as described previously [26, 33]. The genome sequence of the Del4L stock was validated by next-generation sequencing.

### Antibodies and reagents

Rabbit anti-*β*-tubulin (catalog no. A12289), anti-His (catalog no. AE003), mouse anti-HA (catalog no. AE008), anti-Flag (catalog no. AE005), and anti-GST (catalog no. AE001) antibodies were purchased from Abclonal. Rabbit anti-IKK*β* (catalog no. 2678S), rabbit anti-phospho-NF-*κ*B p65 (Ser536) (catalog no. 3033S), rabbit anti-phospho-I*κ*B*α* (Ser32) (catalog no. 2859S), mouse anti-p65 (catalog no. 6956S) and mouse anti-*κ*B*α* (catalog no. 4814S) antibodies were purchased from Cell Signaling Technology. Anti-lamin B1 (catalog no. 12987-1-AP), anti-HSC70 (catalog no. 10654-1-AP), anti-LAMP2A (catalog no. 66301-1-Ig), and mouse anti-GAPDH (60004-1-Ig) antibodies were purchased from Proteintech. HRP-conjugated goat anti-mouse IgG (catalog no. 115-035-003) and goat anti-rabbit IgG (catalog no. 111-035-003) antibodies were purchased from Jackson ImmunoResearch. Alexa Fluor 488-conjugated goat anti-mouse IgG (H + L) (catalog no. A11029), anti-rabbit IgG (H + L) (A11008), and anti-rabbit IgG (H + L) (catalog no. A31573), LICOR IRDye 800CW goat anti-rabbit IgG (H + L) (catalog no. 926-32211) and goat anti-mouse IgG (H + L) (catalog no. 926-32212) antibodies were purchased from Thermo Fisher Scientific. Anti-Flag M2 magnetic beads (catalog no. M8823), anti-Myc affinity gel (Beyotime, catalog no. P2285), and anti-HA agarose (catalog no. A2095) were purchased from Sigma-Aldrich. Anti-A137R, -p72, and -p30 polyclonal antibodies were prepared as described previously [26]. 4’,6-Diamidino-2-phenylindole (DAPI) (catalog no. C006) were purchased from Solarbio. A cell plasma membrane staining kit with DiI (catalog no. C1991S) was purchased from Beyotime. Bafilomycin A1 (BafA1) (catalog no. sc-201550A) was purchased from Santa Cruz. Carbobenzoxy-leu-leu-leucinal (MG132) (catalog no. 474790), lactacystin (catalog no. L6785), and 3-methyladenine (3-MA) (catalog no. M9281) were purchased from Sigma-Aldrich. The recombinant TNF-*α* protein (catalog no. 300-01A-50) was purchased from PeproTech.

### Plasmid construction

To construct the plasmids expressing the HA- or Flag-tagged MGF300-4L, the *MGF300-4L* gene was codon-optimized for expression in HEK293T cells and cloned into the pCMV-HA or pCAGGS-Flag vector by Azenta Life Sciences (Suzhou, China), respectively. The full-length *MGF300-4L* gene was amplified by polymerase chain reaction (PCR) from the ASFV genome and cloned into the pGEX-6P-1 vector (pGST-MGF300-4L). The pGST-MGF300-4L plasmid was transformed in *E. coli* BL21 (DE3) for protein expression. The expression plasmids, including pHis-Ub, pFlag-IKK*α*, pFlag-I*κ*B*α*, pHA-I*κ*B*α*, pFlag-IKK*β*, pHA-IKK*β*, pFlag-NEMO, pNF-*κ*B-Fluc, pTK-Rluc, pHA-NDP52, pHA-OPTN, pHA-p62, and pHA-TOLLIP, were described previously [26]. The pcDNA3.1-myc-*β-*TrCP (PPL00678-2a) and pcDNA3.1-V5-HSC70 (BC007276) plasmids were purchased from Public Protein/Plasmid Library. All plasmids used in this study were verified by sequencing.

### Growth kinetics of ASFV in PAMs

PAMs were grown in 24-well cell culture plates and infected with either Del4L or ASFV-WT at a multiplicity of infection (MOI) of 5 for the single-step growth curve or 0.01 for the multi-step growth curve. The samples were harvested at 2, 12, 24, 48, 72, 96, and 120 hours postinfection (hpi). The virus titers at each time point were determined as described previously [33].

### Laser confocal microscopy

HEK293T cells were grown in glass-bottom culture dishes (NEST, catalog no. 801001) and transfected with the indicated expressing plasmids using X-tremeGENE HP (Roche, catalog no. 6366546001). At 24 hours posttransfection (hpt), the cells were fixed with 4% paraformaldehyde at room temperature for 20 min and washed three times with phosphate-buffered saline (PBS). And then, the samples were permeabilized with 0.1% Triton X-100 (Sigma-Aldrich, catalog no. V900502) in PBS for 20 min. After blocking with 5% bovine serum albumin (BSA), the cells were incubated with the corresponding specific primary antibodies at room temperature for 2 h. After washing with PBS for five times, the cells were stained with fluorescent conjugated secondary antibodies for 1 h. Finally, the cells were incubated with DAPI (nuclei) or DiI (membrane) for 10 min and observed under a laser confocal microscope (LSM880, Zeiss).

### RNA-Seq analysis and quantitative reverse transcription-PCR (RT-qPCR)

For RNA-seq analysis, PAMs were cultured in 24-well cell culture plates and infected with either Del4L or ASFV-WT in triplicates at an MOI of 5 for 12 or 20 h. Subsequently, the cells were lysed in the TRIzol reagent (Invitrogen, catalog no. 15596026CN). RNA-seq and data analysis were performed as described previously [25]. Total RNAs were extracted from the PAMs infected with Del4L or ASFV-WT using an RNA isolation kit (BioFlux, catalog no. BSC52M1) according to the manufacturer’s instructions. For RT-qPCR, cDNA was generated using FastKing gDNA Dispelling RT SuperMix (Tiangen, catalog no. KR118-02) and then amplified by qPCR using SYBR Premix Ex Taq (TaKaRa, catalog no. RR390B) with specific primers (Table S1). The obtained data were normalized to glyceraldehyde-3-phosphate dehydrogenase (GAPDH). The relative abundance of each gene was calculated using the comparative cycle threshold (2^-ΔΔCT^) method.

### Enzyme-linked immunosorbent assay (ELISA)

PAMs were infected with Del4L or ASFV-WT at an MOI of 5. The expression of IL-1*β* (Ray Biotech, catalog no. ELP-IL1b-1), TNF-*α* (Ray Biotech, catalog no. ELP-TNFa-1)*,* and IFN-*α* (Ray Biotech, catalog no. ELP-IFNa-1) in the cell culture supernatants was measured by ELISA kits according to the manufacturer’s protocols at 12 and 20 hpi. The concentrations of these cytokines were determined based on standard curves.

### Luciferase reporter assay

For the luciferase reporter assay, HEK293T cells were cotransfected with pNF-*κ*B-FLuc and pTK-RLuc along with the MGF300-4L expressing plasmids using X-tremeGENE HP. Following stimulation, the cells were lysed, and the luciferase activity was measured by a dual-luciferase reporter assay system (DLR) (Promega, catalog no. E1910) according to the manufacturer's protocols. Luciferase readings were conducted employing the BioTek Synergy plate reader. The luciferase activity was initially normalized to the *renilla* luciferase activity and then further normalized to the non-stimulated empty vector group.

### NF-*κ*B-p65 nuclear translocation assay

HEK293T cells were transfected with the expressing plasmid pFlag-MGF300-4L. At 24 hpt, the cells were treated with 10 ng/mL TNF-*α* for 1 h. The separation of cellular fractions was performed by the Minute plasma membrane protein isolation and cell fractionation kit (Invent Biotechnologies, catalog no. SM-005). The nuclear and cytoplasmic compartments were examined by western blotting, with tubulin and lamin B1 serving as markers for the cytoplasm and nucleus, respectively. To investigate the effects of MGF300-4L on the nuclear translocation of NF-*κ*B-p65 following ASFV infection, PAMs were infected with either ASFV-WT or Del4L (MOI = 5). At 24 hpi, the cells were treated with 10 ng/mL TNF-*α* for 1 h and then the cellular fractions of the ASFV-infected PAMs were separated using the same method as described above.

### Western blotting

Cells were lysed in ice-cold RIPA lysis buffer (Thermo Fisher Scientific, catalog no. 89901) supplemented with a protease inhibitor cocktail and a phosphatase inhibitor cocktail (Roche, catalog no. 4906845001). The resulting cell lysates were then centrifuged at 12,000 rpm at 4°C for 30 min. Total proteins from the cell extracts were resolved by sodium dodecyl sulfate-polyacrylamide gel electrophoresis (SDS-PAGE) and transferred to polyvinylidene difluoride (PVDF) membranes. Following incubation with the primary and secondary antibodies, the membranes were visualized by enhanced chemiluminescence (ECL) (Thermo Fisher Scientific, catalog no. 32106) or an Odyssey two-color infrared fluorescence imaging system (LI-COR).

### Co-immunoprecipitation and GST pulldown assays

The transfected HEK293T cells were cultured in 10-cm dishes and then lysed in 0.9 mL of RIPA lysis buffer for 30 min at 4°C. After the removal of nuclei via low-speed centrifugation and collection of 100 μL as input sample for western blotting, the remaining 800 μL of the lysates were incubated with the appropriate primary antibody, followed by the addition of protein G-sepharose beads (Pierce) and gently rotated at 4°C overnight. To capture the Flag-, Myc-, or HA-tagged target proteins, anti-Flag, -Myc, or -HA-agarose beads were subjected to overnight rotation. The beads were collected and washed three times with 1 mL of pre-cooled RIPA lysis buffer. Then, the proteins were eluted from the beads after boiling in 80 μL of the RIPA lysis buffer plus 20 μL of the sample loading buffer (5×) and then subjected to western blotting.

The glutathione S-transferase (GST) pulldown assay was performed as described previously [26]. Briefly, the purified recombinant GST-MGF300-4L or GST protein was incubated with the lysates from the HEK293T cells transfected with either pFlag-I*κ*B*α* or pFlag-IKK*β*. At 12 hpt, the proteins pulled down by glutathione-sepharose 4B resin (GE Healthcare, catalog no. 17-0756-01) were subsequently analyzed by western blotting.

### RNA interference (RNAi) assay

Small interference RNAs (siRNAs) specifically targeting the human LAMP2A gene and scramble siRNA control were designed and synthesized by GenePharma (Shanghai, China) (Table S1). The HSC70-specific siRNAs (catalog no. sc-29349) were purchased from Santa Cruz Biotechnology. For siRNA transfection, 100 pmol of the siRNAs were transfected into the HEK293T cells or PAMs using Lipofectamine RNAiMAX (Thermo Fisher Scientific, catalog no. 13778030) according to the manufacturer’s instructions. The expression of related proteins was analyzed by western blotting at 24 hpt.

### Pig inoculation experiment

Ten healthy SPF piglets, aged 7 weeks and weighing between 8.5 and 13.25 kg, were obtained from the laboratory animal center of the HVRI. Back titrations were performed to confirm the Del4L or ASFV-WT inoculum titers. The piglets were randomly divided into three groups and inoculated intramuscularly (i.m.) with ASFV to investigate the pathogenicity of the Del4L. Four piglets were inoculated with Del4L (10^2.0^ TCID_50_/piglet); three piglets received inoculation with ASFV-WT (10^2.0^ TCID_50_/piglet); three piglets were inoculated with RPMI 1640 (1 mL/piglet) as a control. Daily monitoring was conducted for each pig, which included recording rectal temperatures and observing the clinical signs of each pig, such as lethargy, anorexia, depression, vomiting, fever, skin hemorrhages, bloody diarrhea, and joint swelling. EDTA-anticoagulated blood and sera were collected from all the pigs before (0) and at 1, 3, 5, 7, 10, 14, and 21 days postinfection (dpi). At 21 dpi, all the piglets that survived were euthanized. From each necropsied pig, seven different tissues and organs (heart, liver, spleen, lung, kidney, submandibular lymph nodes, and mesenteric lymph nodes) were collected for viral load detection using qPCR, following the previously described method [33]. All the pig experiments with ASFV challenge were conducted in the BSL3 facilities at HVRI.

### ASFV-specific antibody inhibition assay and multiplex cytokine assay

The ASFV-specific antibodies against p72 (Ingenasa, catalog no. 11.PPA.K.3/5) and p30 (IDvet, catalog no. ASFC-5P) in the serum samples were measured by ELISA kits. The optical density measured at 450 nm was analyzed using the BioTek Gen5 software. The blocking percentage was calculated according to the equation provided by the manufacturer. For the anti-p72 antibodies, the samples with a blocking percentage above 50% were considered positive, and those below 40% were considered negative. For the anti-p30 antibodies, the samples with a blocking percentage above 50% were considered negative, while below 40% was considered positive. The related cytokines in the collected serum samples, including IL-1*β*, TNF-*α*, and IFN-*α*, were measured by ELISA kits as described above.

### IFN-*γ* enzyme-linked immunospot assay (ELIspot)

To detect ASFV-specific cellular immune responses induced by Del4L infection, ELIspot was performed according to the instructions of a porcine IFN-*γ* ELIspot kit (Mabtech, catalog no. MBT-3130-4HPW-2). Briefly, peripheral blood mononuclear cells (PBMCs) were isolated from EDTA-anticoagulated blood by gradient centrifugation with a porcine peripheral blood lymphocyte isolation kit (Tianjin Haoyang, catalog no. LTS1110). The PBMCs were then cultured in 96-well cell culture plates at a concentration of 5 × 10^5^ cells per well at 37°C with 5% CO_2_ for 20 h. ASFV-WT (10^6.0^ TCID_50_) was used for stimulation, while RPMI 1640 and phytohemagglutinin (PHA) were used as negative and positive controls, respectively. After stimulation with ASFV or PHA, the 96-well cell culture plate was incubated at 37°C with 5% CO_2_ for 20 h. The culture medium was discarded, and the wells were washed five times with PBS. And then, the diluted antibody containing 0.5% FBS was added and incubated at room temperature for 2 h. The diluted streptavidin-HRP containing 0.5% FBS was added and incubated for 1 h after the plate was washed five times with PBS. Finally, the 3,3’,5,5’-tetramethylbenzidine (TMB) substrate solution was added, incubated, and developed until distinct colored spots. The number of spots from each well was then counted by ISpot Spectrum ELR088IFL (Germany).

### Statistical analysis

Data are presented as the mean ± SD, and an unpaired *t* test was used to determine statistical significance with the GraphPad Prism 9. Differences between the two groups were considered significant when the *P* value was less than 0.05.

## Results

### The *MGF300-4L*-deleted ASFV (Del4L) induces a significant production of inflammatory cytokines IL-1*β* and TNF-*α* in PAMs

**Figure 1.**
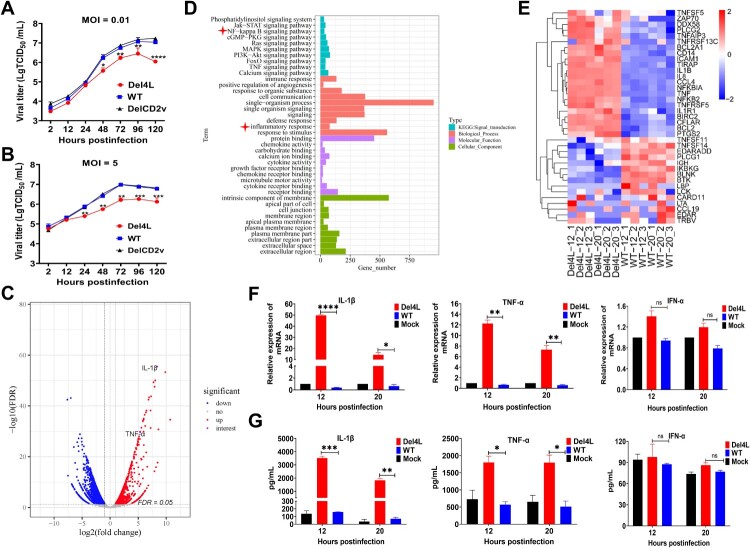
The *MGF300-4L*-deleted ASFV mutant (Del4L) induces higher IL-1*β* and TNF-*α* production than does the wild-type ASFV in primary porcine alveolar macrophages (PAMs). (A and B) Replication characteristics of Del4L in PAMs. PAMs were infected with Del4L, DelCD2v, or the ASFV HLJ/18 strain (ASFV-WT) and the virus yield was titrated at the indicated times and shown as multi-step (MOI = 0.01) (A) or single-step (MOI = 5) (B) growth curve. (C to E) Gene expression profiling in the Del4L-infected PAMs by RNA-seq analysis. PAMs were either mock-infected or infected with Del4L or ASFV-WT (MOI = 5) for 12 and 20 h for RNA-seq analysis. A volcano plot was used to depict the gene expression changes in the Del4L-infected vs the ASFV-WT-infected PAMs (C). Upregulated and downregulated differentially expressed genes (DEGs) were represented by red and blue dots, respectively. Upregulated DEGs were compared between the Del4L and ASFV-WT groups by gene ontology (GO) category functional enrichment analysis. The analysis included three categories: biological process, molecular function, and cell component. Additionally, Kyoto Encyclopedia of Genes and Genomes (KEGG) pathway analysis was performed. The red asterisks indicate the signaling pathways of interest (D). The heatmaps displaying the expression levels of DEGs in the NF-*κ*B signaling pathway induced by Del4L compared with ASFV-WT at 12 and 20 hours postinfection (hpi) (E). (F and G) Del4L induces higher cytokine production than does ASFV-WT in PAMs. PAMs were mock-infected or infected with Del4L or ASFV-WT (MOI = 5). The mRNA levels of IL-1*β*, TNF-*α*, and IFN-*α* (F) in the cell lysates were determined by RT-qPCR at 12 and 20 hpi. The values of the mock-infected group were set to 1 and the values for the infected groups were normalized using those of the mock-infected group. The production of IL-1*β*, TNF-*α*, and IFN-*α* (G) in the cell culture supernatants was detected by commercial ELISA kits at 12 and 20 hpi. * *P* < 0.05; ** *P* < 0.01; *** *P* < 0.001; and ns, not significant (*P* > 0.05).

When screening for the regulation of the ASFV replication-related genes in the LVR, we observed a significant decrease in ASFV replication in PAMs following the deletion of the *MGF300-4L* gene [26]. However, the biological role of MGF300-4L in ASFV infection remains largely uncharacterized. Hence, our focus is to elucidate the precise mechanism by which MGF300-4L regulates ASFV replication. The MGF300-4L protein is a polypeptide comprised of 330 amino acid residues. It exhibits a high degree of conservation across various ASFV strains (Figure S1A) and is primarily located in the cytoplasm (Figure S1B). Additionally, the expression kinetics of the *MGF300-4L* gene was assessed by RT-qPCR. The results showed that the transcription kinetics of *MGF300-4L* was similar to that of the well-characterized early gene *CP204L* (p30) of ASFV (Figure S1C). To further confirm the impact of MGF300-4L on ASFV replication, we compared the replication kinetics between Del4L and ASFV-WT in PAMs, with the *CD2v* gene-deleted virus (DelCD2v) serving as a control. In comparison to ASFV-WT, the Del4L replication was significantly reduced (approximately 10-fold) from 48 to 120 hpi, as shown in both multi-step (Figure 1A) and one-step (Figure 1B) growth curves. As reported, the deletion of the *CD2v* gene does not affect ASFV replication in PAMs [37].

To elucidate the molecular mechanism by which MGF300-4L regulates ASFV replication, we conducted RNA-seq analysis to investigate the host immune responses induced by Del4L infection in PAMs. As shown in Figure 1C, there were 3783 differentially expressed genes (DEGs) between Del4L and ASFV-WT at 12 hpi. Among these, 1224 genes were upregulated, and 2559 ones were downregulated. Similarly, at 20 hpi, we identified 4083 DEGs between Del4L and ASFV-WT, with 1036 genes being upregulated and 3047 ones downregulated (Figure S2A). Significantly, the expression levels of IL-1*β* and TNF-*α* in the Del4L-infected PAMs were approximately 10 and 5 folds higher at both 12 (Figure 1C) and 20 (Figure S2A) hpi, respectively, than those in the ASFV-WT-infected cells. Compared with the ASFV-WT-infected PAMs, the DEGs in the Del4L-infected cells were primarily engaged in innate immunity signaling pathways, including the NF-*κ*B, TNF, MAPK, JAK-STAT, PI3K-AKT, immune response, and inflammatory response signaling pathways (Figure 1D). Additionally, the DEGs were also involved in receptor signaling, defense response, cytokine activity, metabolic networks, and other pathways associated with viral infection (Figure 1D and Figure S2B). Notably, the DEGs from the NF-*κ*B signaling pathway were presented in Figure 1E, wherein IL-1*β*, TNF-*α*, and IL-8 exhibited significantly higher transcription levels in the Del4L-infected PAMs than those in the ASFV-WT-infected PAMs at 12, and 20 hpi.

To further validate the RNA-seq results, the cytokines IL-1*β*, TNF-*α*, and IFN-*α* were measured by RT-qPCR and ELISA. The mRNA expression of IL-1*β* was upregulated by approximately 120 and 15 folds in the Del4L-infected PAMs at 12 and 20 hpi, respectively, compared with ASFV-WT (Figure 1F). Similar to IL-1*β*, the mRNA expression of TNF-*α* was upregulated by approximately 20 and 10 folds at 12 and 20 hpi, respectively, in the Del4L-infected PAMs than in the ASFV-WT-infected cells (Figure 1F). Moreover, the secretion of IL-1*β* and TNF-*α* induced by Del4L infection was increased up to about 22 and 4 folds, respectively, higher than that induced by ASFV-WT infection in PAMs (Figure 1G). However, no significant differences in IFN-*α* levels were found between the PAMs infected with Del4L or ASFV-WT at both the mRNA (Figure 1F) and protein (Figure 1G) levels. Altogether, the obtained results demonstrate that MGF300-4L expressed during ASFV infection inhibits the production of inflammatory cytokines IL-1*β* and TNF-*α* in PAMs.

### The MGF300-4L protein inhibits the activation of the NF-*κ*B signaling pathway

**Figure 2.**
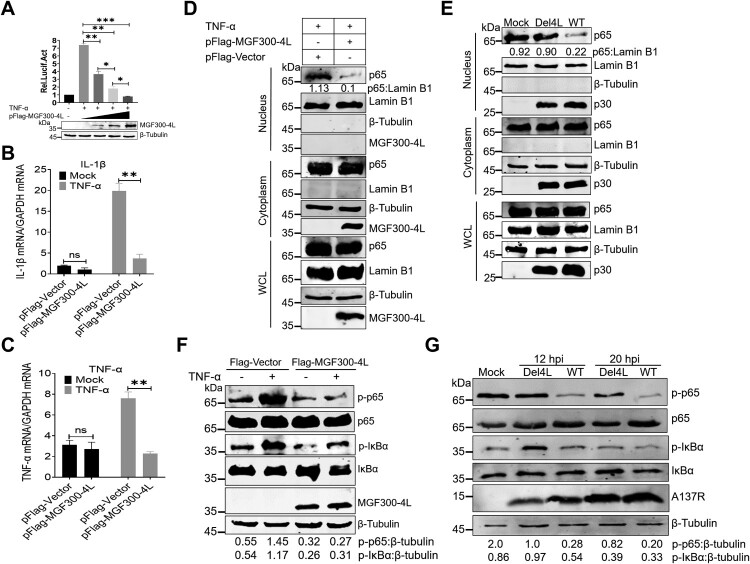
The MGF300-4L protein inhibits the NF-*κ*B signaling pathway. (A to C) The MGF300-4L protein negatively regulates the NF-*κ*B signaling pathway activation. HEK293T cells were cotransfected with the NF-*κ*B reporter plasmids and an increasing amount of pFlag-MGF300-4L (0.1, 0.3, and 0.5 μg). At 24 hours posttransfection (hpt), the cells were either mock-treated or treated with TNF-*α* at a concentration of 10 ng/mL for 6 h. Subsequently, the luciferase activity was measured. The values of the mock-treated group were set to 1 and the values for the TNF-*α*-treated groups were normalized using those of the mock-infected group (A). HEK293T cells were transfected with the empty vector (pFlag-Vector) or pFlag-MGF300-4L. The cells were mock-treated or treated with TNF-*α* at a concentration of 10 ng/mL for 1 h at 24 hpt. The mRNA expression levels of IL-1*β* and TNF-*α* were measured by RT-qPCR (B and C). (D and E) The MGF300-4L protein inhibits the nuclear translocation of p65. HEK293T cells were transfected with pFlag-MGF300-4L or pFlag-Vector and then treated with or without TNF-*α* (10 ng/mL) for 1 h at 24 hpt. The cells were fractionated into cytoplasmic and nuclear fractions and analyzed by immunoblotting (IB) with the indicated antibodies. The p65 in the nuclear and cytoplasmic compartments and the whole cell lysates (WCL) was analyzed by IB. Lamin B1 and *β-*tubulin were selected as markers for the nuclear and cytosolic fractions, respectively (D). PAMs were either mock-infected or infected with ASFV-WT or Del4L at an MOI of 5. At 20 hours postinfection (hpi), the mock-infected and the ASFV-infected PAMs were treated with or without TNF-*α* (10 ng/mL) for 1 h. Subsequently, cellular fractions of the ASFV-infected PAMs were separated following the previously described method [26] (E). (F and G) The MGF300-4L protein inhibits the phosphorylation levels of I*κ*B*α* and p65. HEK293T cells were transfected with pFlag-MGF300-4L or pFlag-Vector and then treated with or without TNF-*α* (10 ng/mL) for 30 min at 20 hpt. The phosphorylation levels of I*κ*B*α* and p65 were analyzed by IB with the indicated antibodies (F). The phosphorylation levels of I*κ*B*α* and p65 in the PAMs mock-infected or infected with ASFV-WT or Del4L (MOI = 5) were analyzed by IB at 12 and 20 hpi (G). * *P* < 0.05; ** *P* < 0.01; *** *P* < 0.001; and ns, not significant (*P* > 0.05).

Transcriptional regulation of inflammatory genes is mediated by transcription factors that are activated through the NF-*κ*B, MAPK, or STAT3 signaling pathways [19]. To gain a more profound comprehension of how MGF300-4L facilitates viral replication by inhibiting inflammatory signaling pathways, a reporter assay was conducted to evaluate the impact of MGF300-4L on NF-*κ*B activation. Our results demonstrated that the ectopically expressed MGF300-4L inhibited the TNF-*α*-triggered activation of the NF-*κ*B promoter in a dose-dependent manner (Figure 2A). Next, we assessed the impacts of MGF300-4L on the mRNA expression levels of IL-1*β* and TNF-*α*. As expected, the overexpression of MGF300-4L significantly suppressed the expression of IL-1*β* (Figure 2B) and TNF-*α* (Figure 2C). Additionally, subcellular fractionation experiments showed that the ectopically expressed MGF300-4L inhibited the nuclear translocation of p65 (Figure 2D). Furthermore, the confocal microscopy showed that MGF300-4L significantly inhibited the TNF-*α*-induced nuclear translocation of p65 (Figure S3). Importantly, we investigated the nuclear localization pattern of p65 in the context of ASFV infection. As shown in Figure 2E, p65 in the nuclear fraction of the Del4L-infected PAMs was significantly increased compared with that of the ASFV-WT-infected PAMs. In summary, these findings indicate that MGF300-4L effectively inhibits the activation of NF-*κ*B by suppressing the nuclear translocation of p65.

The function of MGF300-4L was further investigated by studying its inhibitory effects on two hallmarks of NF-*κ*B activation: phosphorylation of p65 and I*κ*B*α*. The results demonstrated that the ectopically expressed MGF300-4L inhibits p65 and I*κ*B*α* phosphorylation upon stimulation with TNF-*α* (Figure 2F). Moreover, to examine the impacts of MGF300-4L on the phosphorylation of p65 and I*κ*B*α* during virus infection, we infected PAMs with Del4L or ASFV-WT and performed western blotting to detect the phosphorylation levels of p65 and I*κ*B*α*. Consistently, the results showed that infection with Del4L induced higher levels of phosphorylation in p65 and I*κ*B*α* than ASFV-WT infection at 12 and 20 hpi in PAMs (Figure 2G). Collectively, these results suggest that MGF300-4L negatively regulates the production of IL-1*β* and TNF-*α* mediated by the NF-*κ*B signaling pathway.

### The MGF300-4L protein interacts with both IKK*β* and I*κ*B*α*

**Figure 3.**
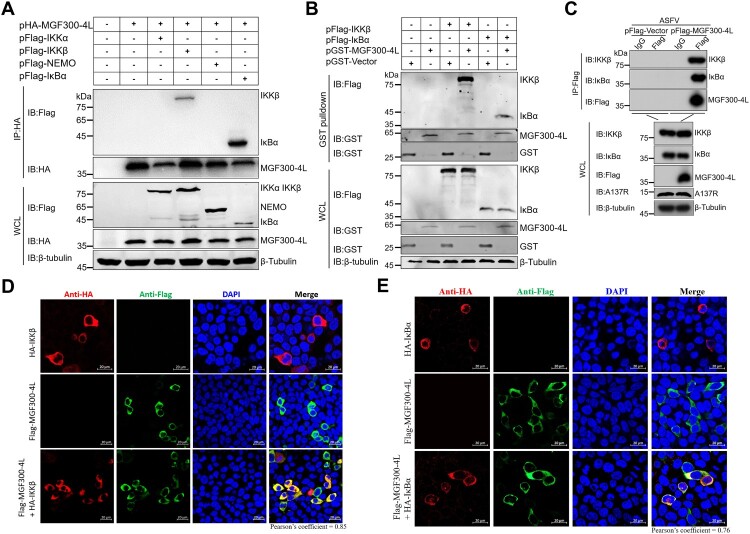
The MGF300-4L protein interacts with both IKK*β* and I*κ*B*α*. (A to C) The MGF300-4L protein interacts with the regulatory proteins of NF-*κ*B. HEK293T cells were transfected with pHA-MGF300-4L along with pFlag-IKK*α*, pFlag-IKK*β*, pFlag-NEMO, or pFlag-I*κ*B*α* as indicated. The cells were lysed and the whole cell lysates (WCL) were immunoprecipitated with an anti-HA monoclonal antibody (mAb) at 36 hours posttransfection (hpt). The immunoprecipitates were examined by immunoblotting (IB) with the indicated antibodies (A). HEK293T cells were transfected with pFlag-IKK*β* or pFlag-I*κ*B*α*. At 36 hpt, the cells were lysed with RIPA buffer. The purified GST or GST-MGF300-4L proteins were used to pull down the IKK*β* or I*κ*B*α* in the lysates and analyzed by IB with the indicated antibodies (B). HEK293T cells were transfected with either pFlag-Vector or pFlag-MGF300-4L. At 20 hpt, the cells were infected with the cell-adapted ASFV for 20 h. Subsequently, the cells were lysed and immunoprecipitation was performed using an anti-Flag or IgG antibody. The WCL and the immunoprecipitates complexes were analyzed by IB (C). (D and E) The ASFV MGF300-4L is colocalized with IKK*β* and I*κ*B*α*. HEK293T cells were transfected with pFlag-MGF300-4L alone or cotransfected with pHA-IKK*β* or pHA-I*κ*B*α* in combination with pFlag-MGF300-4L. IKK*β*, I*κ*B*α*, and MGF300-4L were detected by laser confocal microscopy. The colocalization of MGF300-4L and IKK*β* or I*κ*B*α* was analyzed by the Coloc2 tool of ImageJ/FIJI and shown as Pearson’s correlation coefficients. Scale bar, 20 μm.

The results presented above indicate that MGF300-4L functions at or upstream of I*κ*B*α* in the NF-*κ*B signaling pathway. To elucidate the potential mechanism(s) underlying MGF300-4L function, co-immunoprecipitation (co-IP) of MGF300-4L with various mediators, including IKK*α*, IKK*β*, NEMO, and I*κ*B*α*, of the NF-*κ*B signaling pathway was performed. Co-IP analysis revealed that MGF300-4L specifically co-precipitated with both IKK*β* and I*κ*B*α*, but not with IKK*α* and NEMO (Figure 3A). This interaction was further validated by GST pulldown assay, where the Flag-tagged IKK*β* and I*κ*B*α* were pulled down by GST-MGF300-4L (Figure 3B). In addition, endogenous co-IP assays indicated that MGF300-4L was constitutively associated with IKK*β* and I*κ*B*α* (Figure S4). To address whether this interaction occurred in the context of ASFV infection, co-IP was performed with extracts from HEK293T cells transfected with the plasmid expressing the N-terminally Flag-tagged MGF300-4L under the control of the *MGF300-4L* promoter, and subsequently infected with the cell-adapted ASFV strain. As expected, we found that both IKK*β* and I*κ*B*α* were pulled down with the anti-Flag beads, and immunoblotting of the eluates confirmed the association of MGF300-4L with IKK*β* and I*κ*B*α* (Figure 3C). These data showed that the endogenous MGF300-4L protein expressed in the ASFV-infected cells interacted with IKK*β* and I*κ*B*α*. In agreement with previous findings, the confocal microscopy analysis indicated the colocalization of MGF300-4L with IKK*β* (Figure 3D), as well as MGF300-4L and I*κ*B*α* (Figure 3E), in the cytoplasm of the cells. Overall, these data show that MGF300-4L interacts with both IKK*β* and I*κ*B*α* to counteract the activation of the NF-*κ*B signaling pathway.

### The MGF300-4L protein induces the degradation of IKK*β* via CMA

**Figure 4.**
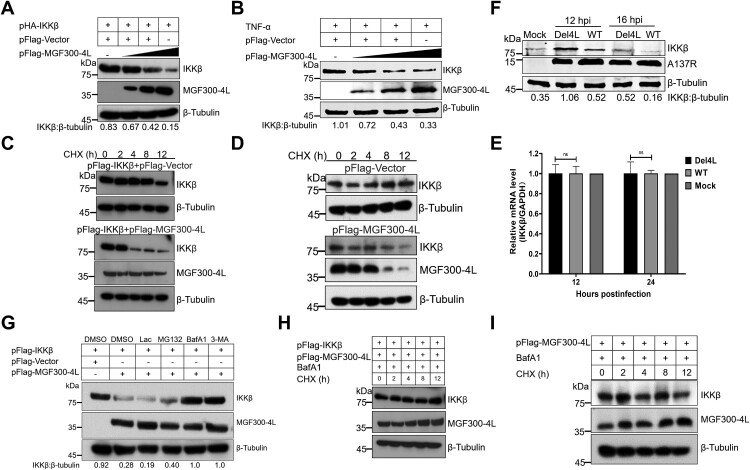
The MGF300-4L protein promotes the autophagic degradation of IKK*β*. (A to B) The effects of MGF300-4L on the expression of IKK*β*. HEK293T cells were cotransfected with pHA-IKK*β* and an increasing amount of pFlag-MGF300-4L (0.5, 1, and 1.5 μg). At 24 hours posttransfection (hpt), the cell lysates were analyzed by immunoblotting (IB) (A). HEK293T cells were transfected with an increasing amount of pFlag-MGF300-4L (0.5, 1, and 1.5 μg). At 24 hpt, the cells were treated with TNF-*α* (10 ng/mL) for 30 min. The expression level of endogenous IKK*β* in the cells was analyzed by IB (B). (C and D) Cycloheximide (CHX) chase assay. HEK293T cells were transfected with pFlag-IKK*β* or cotransfected with pFlag-IKK*β* and pFlag-MGF300-4L, and then the cells were treated with CHX (100 μg/mL) and harvested at the indicated time points. The cell lysates were analyzed by IB (C). HEK293T cells were transfected with pFlag-Vector or pFlag-MGF300-4L, and then the cells were treated with CHX (100 μg/mL) and harvested at the indicated time points. The expression level of endogenous IKK*β* in the cells was analyzed by IB (D). PAMs were either mock-infected or infected with Del4L or ASFV-WT (MOI = 5). At 12 and 24 hours postinfection (hpi), the mRNA level of IKK*β* in the cell lysates was determined by RT-qPCR. The values of the mock-infected group were set to 1 and the values for the infected groups were normalized using those of the mock-infected group (E). (F) The effects of MGF300-4L on the expression of IKK*β* during ASFV infection. PAMs were mock-infected or infected with ASFV-WT or Del4L (MOI = 5) and analyzed for IKK*β* and ASFV-A137R expression levels at 12 and 16 hpi by IB. (G) The effects of inhibitors on the ubiquitin-proteasome and autophagy-lysosome pathways. HEK293T cells were cotransfected with pFlag-IKK*β* and pFlag-MGF300-4L. At 24 hpt, the cells were treatment with Lac (20 μM), MG132 (20 μM), BafA1 (100 nM), or 3-MA (10 mM) for 6 h. The cell lysates were analyzed by IB. (H and I) CHX chase assay. HEK293T cells were cotransfected with pFlag-IKK*β* and pFlag-MGF300-4L (H) or transfected with pFlag-MGF300-4L (I). Afterwards, the cells were treated with BafA1 (100 nM) and CHX (100 μg/mL), and the cell lysates were analyzed by IB at the indicated time points. * *P* < 0.05; ** *P* < 0.01; *** *P* < 0.001; and ns, not significant (*P* > 0.05).

Next, to investigate the impacts of MGF300-4L on the protein level of IKK*β*, we cotransfected HEK293T cells with pFlag-MGF300-4L and pHA-IKK*β*. The results indicated that MGF300-4L inhibited the expression of both exogenous (Figure 4A) and endogenous (Figure 4B) IKK*β* in a dose-dependent manner. To examine the effects of MGF300-4L on the degradation of IKK*β*, a cycloheximide (CHX)-chase assay was conducted. The cells were transfected with pFlag-MGF300-4L, with or without pFlag-IKK*β*. The results demonstrated a significant acceleration in the degradation of the ectopically expressed IKK*β* when MGF300-4L was expressed, compared with the vector transfection group (Figure 4C). We further analyzed the impacts of MGF300-4L on the stability of endogenous IKK*β* by CHX-chase assay. Consistently, the results showed that MGF300-4L accelerated the degradation of endogenous IKK*β* (Figure 4D). Collectively, the data reveal that MGF300-4L promotes the degradation of the both ectopically expressed and endogenous IKK*β*. And we found that increased ectopically expression of MGF300-4L did not affect the transcriptional level of IKK*β* (Figure S5). In addition, no significant differences were observed in the endogenous mRNA level of IKK*β* in PAMs infected with ASFV-WT or Del4L at 12 and 24 hpi (Figure 4E). Furthermore, the degradation of endogenous IKK*β* was less pronounced in the Del4L-infected PAMs than in the ASFV-WT-infected cells at 12 and 16 hpi (Figure 4F). These observations suggest that the absence of endogenously expressed MGF300-4L during ASFV infection leads to increased IKK*β* levels in PAMs. This further confirms the role of MGF300-4L in inhibiting the NF-*κ*B-mediated innate immune response by promoting the degradation of IKK*β*.

The ubiquitin-proteasome system (UPS) and autophagy are crucial intracellular protein degradation pathways in eukaryotic cells [35]. To elucidate the mechanism of the MGF300-4L-mediated IKK*β* degradation, HEK293T cells were cotransfected with pFlagMGF300-4L and pFlag-IKK*β* and treated with Lac and MG132, which block UPS, and BafA1 and 3-MA, which block autophagic degradation. Among these inhibitors, the most significant counteraction to the reduction of IKK*β* was observed by BafA1 and 3-MA, but not by Lac and MG132 (Figure 4G). In accordance with the above finding, the CHX-chase experiment demonstrated a restoration in the degradation of both the ectopically expressed (Figure 4H) and endogenous (Figure 4I) IKK*β* by MGF300-4L upon BafA1 treatment. Together, these results suggest that MGF300-4L promotes the degradation of IKK*β* through the lysosomal pathway.

**Figure 5.**
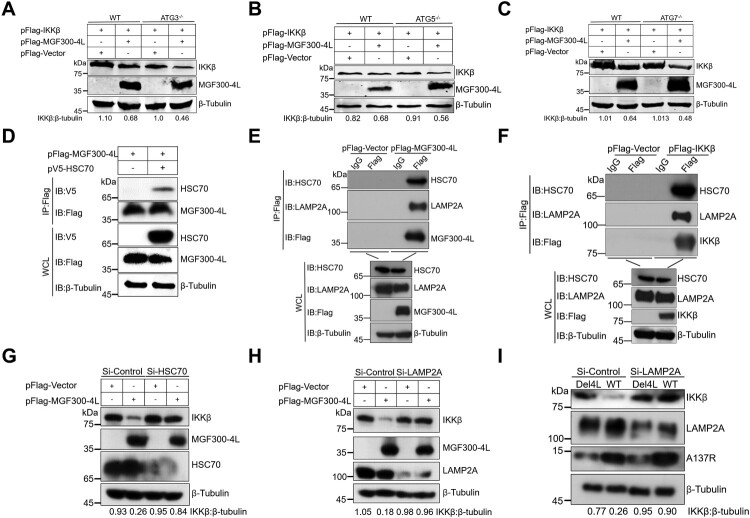
MGF300-4L degrades IKK*β* via the chaperon-mediated autophagy. (A to C) MGF300-4L degrades IKK*β* independently of macroautophagy. pFlag-IKK*β* and pFlag-MGF300-4L were cotransfected into the autophagy-related protein 3-knockout (*ATG^3-/-^*) (A), *ATG^5-/-^* (B), *ATG^7-/-^* (C), or wild-type (WT) HEK293T cells. At 36 hours posttransfection (hpt), the expression levels of IKK*β* and MGF300-4L were analyzed by immunoblotting (IB). (D) MGF300-4L interacts with HSC70. HEK293T cells were cotransfected with pFlag-MGF300-4L along with pV5-HSC70. The cells were lysed and the whole cell lysates (WCL) were immunoprecipitated with an anti-Flag monoclonal antibody. The immunoprecipitates were examined by IB with the indicated antibodies. (E and F) MGF300-4L or IKK*β* interacts with HSC70 or LAMP2A. HEK293T cells were transfected with pFlag-MGF300-4L or pFlag-IKK*β*. At 24 hpt, the cells were lysed and immunoprecipitated by anti-Flag or IgG antibodies. The WCL and the immunoprecipitates complexes were analyzed by IB with the indicated antibodies. (G to I) MGF300-4L degrades IKK*β* via CMA. HEK293T cells were transfected with pFlag-MGF300-4L and scramble or HSC70- (G) or LAMP2A-specific siRNAs (H). At 24 hpt, the protein levels of MGF300-4L, endogenous IKK*β*, HSC70, and LAMP2A were checked by IB. PAMs were transfected with scramble or LAMP2A-specific siRNAs for 20 h. The protein levels of A137R, endogenous IKK*β*, and endogenous LAMP2A were checked by IB (I).

To further dissect the mechanism involved in the MGF300-4L-mediated IKK*β* degradation, we performed an analysis using cell lines in which autophagy-related genes (ATG) were knocked out. The results indicated that the degradation of IKK*β* by MGF300-4L is independent of ATG3 (Figure 5A), ATG5 (Figure 5B), and ATG7 (Figure 5C). Our current and previous studies suggest that the virus-encoded protein has the ability to recruit autophagy receptor p62 or TOLLIP for the degradation of IKK*β* via the lysosomal pathway [26]. Furthermore, the co-IP results revealed that MGF300-4L interacted with the cargo receptor TOLLIP (Figure S6A). However, subsequent results indicated that the degradation of IKK*β* by MGF300-4L is also independent of p62 (Figure S6B) or TOLLIP (Figure S6C). Based on the aforementioned results, we presume that MGF300-4L induces degradation of IKK*β* via CMA or microautophagy. It has been shown that LAMP2A and HSC70 play integral roles in the chaperone-mediated autophagy (CMA): HSC70 identifies and interacts with the CMA motif of target proteins, while LAMP2A, which is located in the lysosomal membrane, facilitates the transportation of the target proteins into lysosomes for degradation [30]. Next, we investigated whether CMA was involved in the selective degradation of IKK*β* by MGF300-4L. Co-IP analysis revealed that MGF300-4L interacted with HSC70 (Figure 5D). In addition, endogenous co-IP assays indicated that MGF300-4L and IKK*β* were constitutively associated with HSC70 and LAMP2A (Figure 5E,F). Importantly, the siRNAs-mediated knockdown of HSC70 (Figure S6D) or LAMP2A (Figure S6E) expression prevented MGF300-4L-mediated ectopically expressed IKK*β* degradation. Consistently, the degradation of endogenous IKK*β* by MGF300-4L could also be restored upon knockdown of HSC70 (Figure 5G) or LAMP2A (Figure 5H) by RNAi. Furthermore, the MGF300-4L-mediated degradation of endogenous IKK*β* was restored when LAMP2A was knocked down by RNAi in PAMs in the context of ASFV infection (Figure 5I). Taken together, these results demonstrated that MGF300-4L induced the lysosomal degradation of IKK*β* via CMA.

### The MGF300-4L protein competes with I*κ*B*α* for binding to *β*-TrCP and thereby blocking I*κ*B*α* degradation

**Figure 6.**
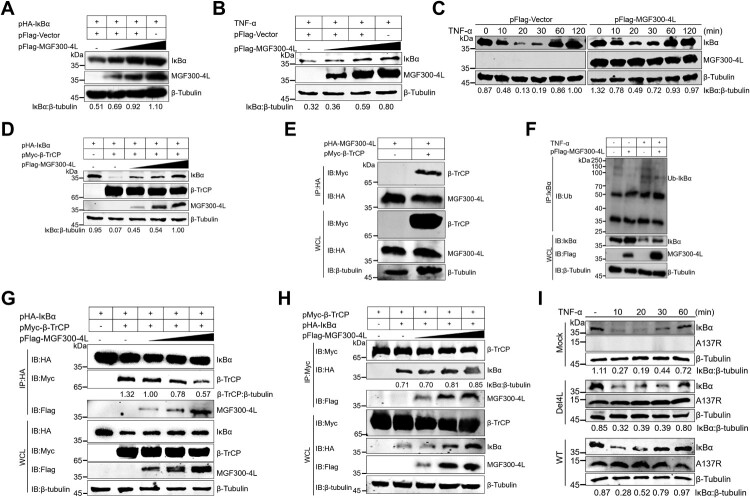
The MGF300-4L protein competes with I*κ*B*α* for binding to *β*-TrCP and thereby blocks I*κ*B*α* degradation. (A to D) The effects of MGF300-4L on the expression of I*κ*B*α*. HEK293T cells were cotransfected with pHA-I*κ*B*α* and an increasing amount of pFlag-MGF300-4L (0.5, 1, and 1.5 μg). At 24 hours posttransfection (hpt), the cell lysates were analyzed for the expression of I*κ*B*α* by immunoblotting (IB) (A). HEK293T cells were transfected with an increasing amount of pFlag-MGF300-4L (0.5, 1, and 1.5 μg). At 24 hpt, the cells were treated with TNF-*α* (10 ng/mL) for 30 min. The cell lysates were analyzed for the expression of endogenous I*κ*B*α* by IB (B). HEK293T cells were transfected with pFlag-MGF300-4L or the empty vector (pFlag-Vector). At 20 hpt, the cells were treated with 10 ng/mL TNF-*α* for the indicated times. The cells lysates were analyzed for I*κ*B*α* expression by IB (C). HEK293T cells were cotransfected with pHA-I*κ*B*α*, pMyc-*β*-TrCP, and an increasing amount of pFlag-MGF300-4L (0.5, 1, and 1.5 μg). At 24 hpt, the cell lysates were analyzed for the ubiquitination degradation of I*κ*B*α* by IB (D). (E) MGF300-4L interacts with *β*-TrCP. HEK293T cells were cotransfected with pHA-MGF300-4L along with pMyc-*β*-TrCP. The cells were lysed and the whole cell lysates (WCL) were analyzed by immunoprecipitation (IP) assay with an anti-HA monoclonal antibody (mAb) at 36 hpt. The immunoprecipitates were examined by IB with the indicated antibodies. (F) MGF300-4L inhibits the ubiquitination of I*κ*B*α*. HEK293T cells were transfected with pFlag-MGF300-4L and then treated with or without TNF-*α* (10 ng/mL) for 30 min. At 24 hpt, the cells were examined for IP with an anti-I*κ*B*α* antibody. The WCL and precipitated proteins were analyzed by IB with the indicated antibodies. (G) The MGF300-4L protein competes with I*κ*B*α* for binding to *β*-TrCP. HEK293T cells were cotransfected with pHA-I*κ*B*α*, pMyc-*β*-TrCP, and an increasing amount of pFlag-MGF300-4L (0.5, 1, and 1.5 μg). At 24 hpt, the cell lysates were used for IP with an anti-HA antibody, followed by IB with the indicated antibodies. (H) I*κ*B*α* does not compete with MGF300-4L for binding to *β*-TrCP. HEK293T cells were cotransfected with pHA-I*κ*B*α*, pMyc-*β*-TrCP, and an increasing amount of pFlag-MGF300-4L (0.5, 1, and 1.5 μg). At 24 hpt, the cell lysates were used for IP with an anti-Myc antibody, followed by IB with the indicated antibodies. (I) MGF300-4L inhibits the degradation of I*κ*B*α* during viral infection. The I*κ*B*α* level in the PAMs mock-infected or infected with ASFV-WT or Del4L (MOI = 5) followed by treatment with or without TNF-*α* (10 ng/mL) were analyzed by IB at the indicated time points.

To investigate the impacts of MGF300-4L on the expression of I*κ*B*α*, we observed a dose-dependent increase in the ectopically expressed I*κ*B*α* in response to MGF300-4L (Figure 6A). To verify the data, we overexpressed MGF300-4L and checked the stability of endogenous I*κ*B*α* in the cells treated with TNF-*α*. The results showed that MGF300-4L increased the expression level of endogenous I*κ*B*α* (Figure 6B). We speculate that MGF300-4L inhibits the ubiquitination degradation of I*κ*B*α*. To verify this hypothesis, we examined the I*κ*B*α* degradation upon MGF300-4L overexpression. The degradation of I*κ*B*α* induced by TNF-*α* in HEK293T cells was markedly suppressed by the expression of MGF300-4L (Figure 6C). The activation of NF-*κ*B requires the phosphorylation of I*κ*B*α* on the serines of positions 32 and 36 within a short motif (DSGX2-3S) [20]. Once this motif is phosphorylated, it is recognized and ubiquitinated by the E3 ligase *β*-TrCP, consequently leading to degradation via the proteasome pathway [20]. Indeed, the expression of MGF300-4L suppressed *β*-TrCP-mediated ubiquitination degradation of I*κ*B*α* in a dose-dependent manner (Figure 6D). Furthermore, the co-IP results also indicate that MGF300-4L interacts with *β*-TrCP (Figure 6E). However, this interaction does not affect the stability of *β*-TrCP, and vice versa (Figure S7A and B). To examine whether MGF300-4L can inhibit the ubiquitination of I*κ*B*α*, we conducted an IP experiment. The results demonstrated that the expression of MGF300-4L suppressed the TNF-*α*-induced ubiquitination of I*κ*B*α* (Figure 6F).

To address whether MGF300-4L competes with I*κ*B*α* for *β*-TrCP binding, MGF300-4L, *β*-TrCP, and I*κ*B*α* were expressed in HEK293T cells, followed by an examination of the interaction of *β*-TrCP with I*κ*B*α* and MGF300-4L. The results showed that MGF300-4L reduced the binding of *β*-TrCP to I*κ*B*α* in a dose-dependent manner (Figure 6G). On the contrary, increased expression of MGF300-4L did not affect the binding of I*κ*B*α* to *β*-TrCP (Figure 6H). Additionally, we observed that ASFV-WT infection inhibited the ubiquitination degradation of I*κ*B*α* induced by TNF-*α* in PAMs (Figure 6I). Importantly, Del4L infection decreased the ubiquitination-mediated degradation of I*κ*B*α* induced by TNF-*α* in contrast with ASFV-WT infection (Figure 6I). These data show that the interaction between MGF300-4L and I*κ*B*α* competitively inhibits the binding of *β*-TrCP to I*κ*B*α*, thereby inhibiting the ubiquitination and degradation of I*κ*B*α*.

### The MGF300-4L protein is associated with ASFV pathogenicity

**Figure 7.**
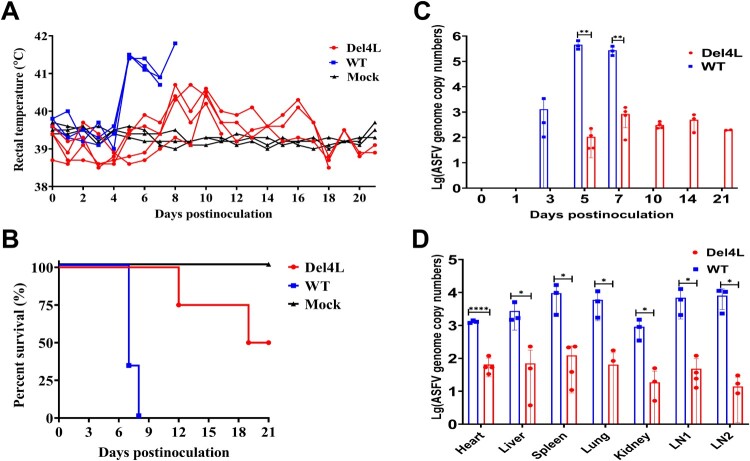
The MGF300-4L protein is associated with the virulence of ASFV. (A to D) Effects of MGF300-4L on ASFV pathogenicity in pigs. The specific-pathogen-free pigs were inoculated intramuscularly (i.m.) with 10^2.0^ TCID_50_ of Del4L (*n* = 4) or ASFV-WT (*n* = 3) or mock-inoculated i.m. with RPMI 1640 (*n* = 3). The rectal temperatures (A) and survival rates (B) of different groups were recorded. (C and D) Viral loads in the inoculated pigs. Genomic copies in the whole blood (C) and different tissues (D) of the pigs inoculated with ASFV-WT or Del4L were detected by qPCR. LN1: submandibular lymph nodes, LN2: mesenteric lymph nodes. * *P* < 0.05; ** *P* < 0.01; *** *P* < 0.001; and ns, not significant (*P* > 0.05).

To further understand the role of MGF300-4L in ASFV pathogenicity, 7-week-old SPF piglets were either mock-infected (*n* = 3) or infected with ASFV-WT (*n* = 3) or Del4L (*n* = 4) at a dose of 10^2.0^ TCID_50_. Clinical signs of the disease related to ASF and survival were monitored daily until 21 dpi. In the ASFV-WT infected group, all three pigs developed a fever at 5 dpi and gradually increased to 41.5°C until their deaths occurred within 8 dpi (Figure 7A). In contrast, all the pigs in the mock group exhibited no obvious ASF-specific clinical signs and maintained normal body temperatures during the 21-d observation period. However, two pigs inoculated with Del4L developed a fever at 8-10 dpi and died at 12 and 19 dpi, respectively (Figure 7B), while the remaining two pigs either maintained a normal body temperature or experienced a transient low fever before returning to normal. The mortality rates for pigs inoculated with ASFV-WT or Del4L was 100% or 50%, respectively (Figure 7B). The viral genomic DNA in the blood of the pigs inoculated with ASFV-WT was detected as early as 3 dpi (1.3 × 10^3.0^ copies/mL), and gradually increased (4.5 × 10^5.0^ copies/mL) until all pigs died. In contrast, the pigs infected with Del4L showed significantly reduced levels of viral genomic DNA in their blood compared with those infected with ASFV-WT (Figure 7C). The tissue samples collected from the heart, liver, spleen, lungs, kidneys, and submaxillary and inguinal lymph nodes of the pigs infected with ASFV-WT showed high viral loads (3.4 × 10^2.0^ to 1.6 × 10^4.0^ copies/mL). In contrast, the tissue samples from the Del4L-inoculated pigs exhibited viral genome copies significantly lower (1/100 to 1/1000) than those in the ASFV-WT-inoculated group (Figure 7D). The data collectively indicate that MGF300-4L is a novel virulence-associated factor of ASFV. Furthermore, the deletion of the *MGF300-4L* gene leads to a significant reduction in the replication of Del4L, both *in vitro* and *in vivo*.

### The wild-type and *MGF300-4L*-deleted ASFV induce different innate and adaptive immune responses *in vivo*

**Figure 8.**
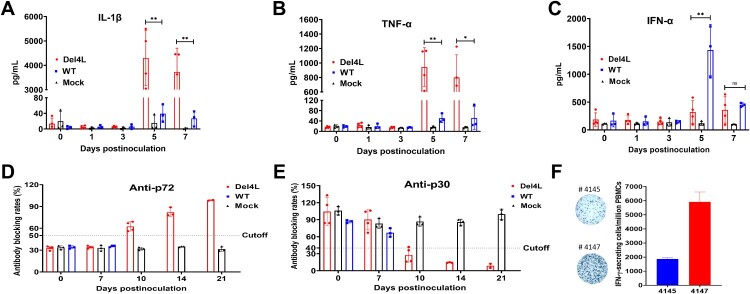
The *MGF300-4L* gene deletion results in pronounced innate and adaptive immune responses to ASFV infection in pigs. (A to C) Del4L infection induces a strong IL-1*β* and TNF-*α* production in pigs. The expression levels of IL-1*β* (A), TNF-*α* (B), and IFN-*α* (C) in the serum samples were measured by commercial ELISA kits. (D and E) Del4L infection induces robust anti-ASFV antibodies in pigs. Serum antibodies against p72 (D) and p30 (E) in the inoculated pigs were detected by ELISA. The results were shown as blocking percentages. The serum sample was regarded as positive when the blocking rate for anti-p72 antibodies exceeded 50% and as negative when the blocking rate below 40%. The sample was regarded as positive for anti-p30 antibodies when the blocking rate was below 40% and as negative when the blocking rate exceeded 50%. The samples exhibiting a blocking rate between 40 and 50% were regarded as doubtful. (F) Del4L infection induces a robust cellular immune response in pigs. The numbers of IFN-*γ*-producing cells in the peripheral blood mononuclear cells (PBMCs) were measured in the two surviving pigs by enzyme-linked immunospot assay (ELIspot). * *P* < 0.05; ** *P* < 0.01; *** *P* < 0.001; and ns, not significant (*P* > 0.05).

Further investigation into the molecular mechanisms through which MGF300-4L influences ASFV pathogenicity is crucial following its identification as a virulence factor. The host innate and adaptive immune responses are well-documented to effectively restrict viral replication. Therefore, we employed ELISA to assess the levels of IL-1*β*, TNF-*α*, and IFN-*α* to analyze the different innate immune responses triggered by Del4L or ASFV-WT infection in pigs. The pigs infected with Del4L produced significantly higher levels of IL-1*β* (Figure 8A) and TNF-*α* (Figure 8B) than did those infected with ASFV-WT at 5 and 7 dpi. In contrast, the ASFV-WT-inoculated pigs exhibited significantly increased levels of IFN-*α* at 5 dpi (Figure 8C). These results demonstrate that the partially attenuated Del4L induces a higher proinflammatory response in pigs than does ASFV-WT.

To clarify the humoral immune responses in the pigs infected with Del4L, we examined the serum anti-p72 and anti-p30 antibodies by ELISA kits (a blocking rate above 50% or below 40% is considered positive for anti-p72 or anti-p30 antibodies, respectively). The anti-p72 (Figure 8D) and anti-p30 (Figure 8E) antibodies in the Del4L-infected pigs showed an increase from 10 dpi and reached peak titers at 21 dpi, indicating that the partially attenuated Del4L induced superior antibody responses. It is noteworthy that Del4L has the capability to rapidly stimulate a humoral immune response, as evidenced by its ability to induce the production of ASFV-specific antibodies as early as 10 dpi. In contrast, the sera collected from the mock and ASFV-WT inoculated pigs showed no presence of anti-p72 and anti-p30 antibodies (Figure 8D,E). To elucidate cellular immune responses in the pigs infected with Del4L, we aimed to identify the cells secreting IFN-*γ* by ELIspot. Importantly, a significant number of ASFV-specific T cells were detected in the two surviving pigs (Figure 8F). The induction of ASFV-specific T cells and antibody responses demonstrated that Del4L is partly attenuated and can induce a systemic immune response *in vivo*. Taken collectively, these findings support our hypothesis that the suppression of host innate responses by MGF300-4L is involved in ASFV pathogenicity.

## Discussion

Identifying virulence determinants of ASFV and elucidating their underlying mechanisms are essential for developing vaccines and novel strategies against ASF [5,10,37]. In this study, MGF300-4L was identified for the first time as a virulence determinant of ASFV due to its dual-target inhibitory effects on the NF-*κ*B signaling pathway. This discovery uncovered a previously unrecognized function of how the canonical NF-κB pathway is finely regulated by MGF300-4L, which greatly helped us understand the virus-host interaction during ASFV infection.

The NF-*κ*B signaling pathway plays a fundamental role in regulating the host's innate immune response, especially in the production of IFNs and inflammatory cytokines IL-1*β* and TNF-*α* [38]. It is not surprising that viruses have evolved various immunoevasion strategies to escape the NF-*κ*B signaling pathway, including Epstein–Barr virus (EBV), VACV, human immunodeficiency virus (HIV), and Kaposi sarcoma herpesvirus (KSHV) [38-40]. It is well established that the antiviral immune response, particularly involving IFNs and inflammatory reactions, significantly contributes to the suppression of viral replication [41]. To investigate the role of MGF300-4L in regulating ASFV replication, we conducted an RNA-seq analysis. Inflammatory cytokines (such as IL-1*β* and TNF-*α*) in the PAMs infected with Del4L showed a significant increase compared with ASFV-WT infection at 12 and 20 hpi. Moreover, the signaling pathways responsible for the production of inflammatory cytokines, including the NF-*κ*B and MAPK pathways, exhibited an enrichment of a substantial number of DEGs. These results were further confirmed through the RT-qPCR and ELISA assays (Figure 1F,G). Notably, there is a significant decrease in the production of IL-1*β* and TNF-*α* at 20 hpi compared with that at 12 hpi, which may be due to the extensive proliferation of ASFV and its cytopathic effects on the cells over time. However, the expression of IFN-*α* did not exhibit any significant differences. Based on the analysis above, we hypothesize that MGF300-4L may exert its function by inhibiting the NF-*κ*B signaling pathway. We conducted a series of biochemical analyses to validate this hypothesis and made remarkable discoveries. We found that the NF-*κ*B-responsive promoter could be inhibited in the presence of MGF300-4L. Moreover, we demonstrated that IL-1*β* and TNF-*α* transcripts were downregulated in the presence of MGF300-4L in an NF-*κ*B-dependent manner. Upon TNF-*α* induction, MGF300-4L significantly reduced the nuclear translocation and phosphorylation of p65. Importantly, these findings have been validated in the context of ASFV infection.

Despite previous reports on NF-*κ*B inhibitors encoded by ASFV, a comprehensive investigation into their mechanisms of action *in vitro* and *in vivo*, specifically regarding viral infection and pathogenicity, has not been detailed [42,43]. To investigate the molecular mechanism by which MGF300-4L inhibits the production of IL-1*β* and TNF-*α*, we performed a protein–protein interaction analysis, which revealed the interactions between MGF300-4L and IKK*β*, as well as the interactions between MGF300-4L and I*κ*B*α* (Figure 3). The further analysis unveiled that the interaction of MGF300-4L with IKK*β* results in the autophagic degradation of IKK*β*. The immune regulatory protein of ASFV degrades key molecules involved in the IFNs and NF-*κ*B signaling pathway via the autophagy pathway, a commonly employed immunoevasion mechanism by ASFV [44]. For example, the virulence-associated factor A137R protein of ASFV can inhibit the production of IFNs by mediating the autophagy-induced lysosomal degradation of TBK1 [45]. The ASFV-encoded L83L protein recruits TOLLIP to promote the autophagic degradation of STING, thereby inhibiting the production of IFNs [46]. The virulence-related factor MGF300-2R protein of ASFV recruits TOLLIP to interact with IKK*α* and IKK*β* (IKK*α*/*β*)*,* thereby promoting the autophagic degradation of IKK*α*/*β*, leading to the inhibition of inflammatory cytokine production [26]. Unlike MGF300-2R, which degrades IKK*α*/*β* through the TOLLIP-mediated selective autophagy, MGF300-4L degrades IKK*β* independent of macroautophagy. Considering that MGF300-4L does not rely on ATG3, ATG5, ATG7, or selective autophagy receptors p62 and TOLLIP for IKK*β* degradation (Figure 5A–C and Figure S6B and C), we speculate that MGF300-4L may degrade IKK*β* through CMA. Based on co-IP assays, we found that MGF300-4L interacted with HSC70 and LAMP2A. The knockdown of LAMP2A restored the MGF300-4L-induced lysosomal degradation of IKK*β*. Hence, we propose that the ASFV MGF300-4L interacts with HSC70 and recruits HSC70 to IKK*β*, thereby promoting the lysosomal degradation of IKK*β* via CMA. These findings have the potential to enhance a better understanding of the role of CMA in the pathogenicity of ASFV. Future experiments will be required to elucidate the precise relationship between MGF300-4L and MGF300-2R.

To our knowledge, MGF300-4L is the first ASFV-encoded protein identified to target I*κ*B*α*. The interaction of MGF300-4L with I*κ*B*α* hinders the *β-*TrCP-mediated ubiquitin degradation of I*κ*B*α*, consequently suppressing NF-*κ*B activation. This mechanism of action differs from several viral proteins that stabilize I*κ*B*α* through molecular mimicry, accomplished by interacting with *β*-TrCP. Notable examples include the Vpu protein of HIV [40], the A49 protein of VACA [20], and the NSP1 protein of rotavirus, which possess a phosphodegron-like (PDL) motif [47], allowing them to interact with *β-*TrCP and subsequently inhibit the *β-*TrCP-mediated degradation of I*κ*B*α* through ubiquitination. It is noteworthy that while MGF300-4L interacts with *β-*TrCP, it cannot bind to *β*-TrCP through molecular mimicry because MGF300-4L lacks a PDL motif. The stabilization of I*κ*B*α* by MGF300-4L is mediated through its direct interaction with I*κ*B*α*, preventing the binding of *β*-TrCP to I*κ*B*α*. This inhibitory effect is likely attributed to MGF300-4L binding to the shared site of *β*-TrCP and I*κ*B*α*.

The analysis of the complete genome sequences has revealed large deletions, including *MGF505* (*1R*-*3R*) and *MGF360* (*10L*-*14L*), in the genome of the attenuated ASFV strains OURT88/3 and NHP68 [48]. Additionally, we and others have also identified large deletions, including *MGF300*, *MGF505*, and *MGF360*, in the LVR of the ASFV genome during the virus adaptation to the cell lines [34,49,50]. Subsequent studies have confirmed that these deleted *MGF505* and *MGF360* genes are involved in ASFV infection and pathogenicity [12,51–54]. However, the role of the *MGF300*-encoded proteins in regulating ASFV replication and pathogenicity remains unclear. In this study, we investigated the relevance of MGF300-4L to the virus pathogenicity in pigs by using a recombinant ASFV (Del4L) lacking the *MGF300-4L* gene. Deleting the *MGF300-4L* gene from the virulent ASFV strain led to a partially attenuated phenotype, resulting in a 50% survival rate (Figure 7B) among the infected pigs and a decreased viral replication in both blood and tissues. Despite two pigs infected with Del4L succumbed to the disease at 12 and 19 dpi. However, their survival time was significantly longer than that of the pigs infected with ASFV-WT (8 dpi). The viral replication of Del4L and ASFV-WT in PAMs and pigs showed significant differences, suggesting that the attenuation of Del4L was mainly due to the reduced viral growth. The pigs infected with Del4L showed elevated levels of IL-1*β* and TNF-*α* at 5 and 7 dpi compared with those infected with ASFV-WT (Figure 8A,B). These findings suggest that the reduced replication of Del4L is mainly attributed to the increased expression of proinflammatory cytokines. Compared with the Del4L-infected pigs, the ASFV-WT-infected pigs exhibited higher levels of IFN-*α* production, which may be attributed to the differences in viral replication. Furthermore, the pigs infected with Del4L can produce ASFV-specific humoral and cellular immune responses. Notably, MGF300-4L is among several ASFV proteins including, H240R, MGF505-7R, and MGF300-2R, all of which inhibit NF-*κ*B activation. When deleted individually from ASFV, they cause an attenuation phenotype *in vivo* [13,26,27]. Intuitively, it seems unlikely that the attenuation would occur due to the loss of a single NF-*κ*B inhibitor, especially when other inhibitors are still present. Remarkably, ASFV lacking the *MGF300-4L* gene, despite encoding additional NF-*κ*B inhibitors, exhibited decreased virulence *in vivo*. One possible explanation for this paradox is that these NF-*κ*B inhibitors encoded by ASFV serve multiple biological functions. In contrast, other functions are not compensated through the remaining NF-*κ*B inhibitors *in vivo*. In this context, immune modulation mediated by MGF300-4L in ASFV can, at least in part, explain why the virulence of Del4L is different from that of ASFV-WT.

A comparison of the pathogenic effects of MGF300-2R and MGF300-4L on ASFV is particularly interesting. When pigs were infected with a dose of 10^2.0^ TCID_50_, the survival rate of the pigs infected with the MGF300-2R gene-deleted ASFV (Del2R) was 100% [26]. In contrast, the survival rate of the pigs infected with the Del4L was 50%. This finding suggests that although the two genes belong to the same MGF, their contributions to the virulence of ASF are different. The possible explanation is that MGF300-2R targets IKK*β* and IKK*α* simultaneously, exerting inhibitory effects on both the classical and non-classical NF-*κ*B signaling pathways. However, MGF300-4L targets two crucial molecules, IKK*β* and I*κ*B*α*, in the classical NF-*κ*B signaling pathway. Both IKK*β* and IKK*α* play crucial roles as converging points for multiple signaling pathways. Thus, the simultaneous targeting of these two molecules may result in a more potent inhibitory effect on antiviral immune responses. However, it cannot be dismissed that MGF300-2R may possess multiple functions. Consequently, the combined action of these viral inhibitors effectively inhibits the NF-*κ*B signaling pathway, with each inhibitor making its unique contribution to the overall virulence of the virus [22].

In conclusion, the multifunctional protein MGF300-4L, encoded by ASFV, exhibits a dual role by degrading IKK*β* through chaperone-mediated autophagy and stabilizing I*κ*B*α* (Figure S8). Hence, it inhibits the production of NF-*κ*B-regulated inflammatory cytokines IL-1*β* and TNF-*α in vitro* and *in vivo*, promoting immunoevasion and enhancing ASFV virulence.

## Supplementary Material

Supplemental Material

Supporting_information_updated_V2_1

Supplementary_Table1

## Data Availability

The RNA sequence reads generated in this study have been deposited in the sequence read archive database under the accession number PRJNA970972 (BioProject). The complete genome sequence of the ASFV HLJ/18 strain used in this study can be accessed under the GenBank accession no. MK333180.1.
