# Supplementary figures and images for: The African swine fever virus MGF300-4L protein is associated with viral pathogenicity by promoting the autophagic degradation of IKKβ and increasing the stability of IκBα
Source: Emerg Microbes Infect. 2024 Mar 19;13(1):2333381. doi: 10.1080/22221751.2024.2333381 (PMC11018083; doi:10.1080/22221751.2024.2333381)

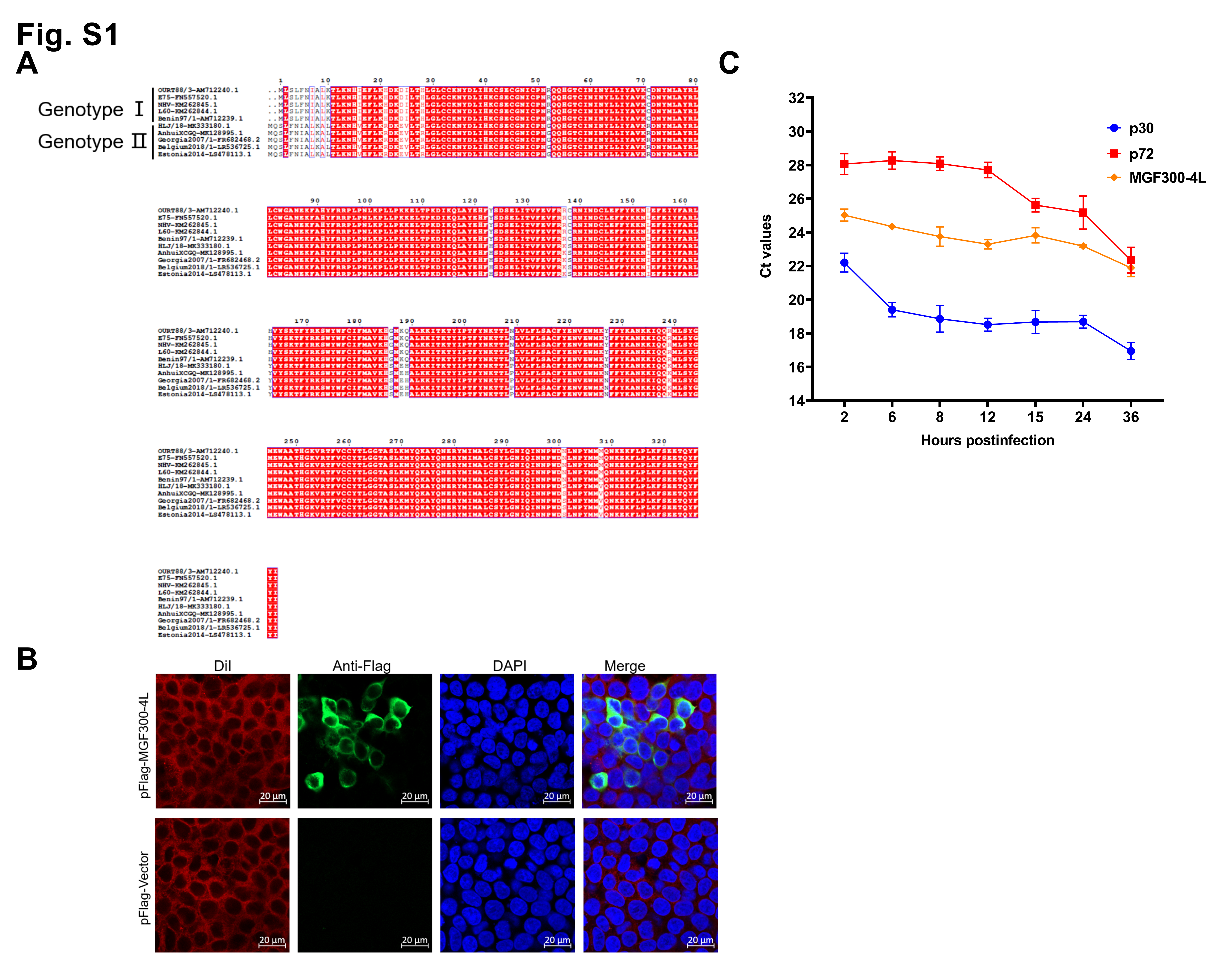

Supplement: Supplemental Material [file TEMI_A_2333381_SM3337.zip › Supplementary_Figures/FigS1.tif]

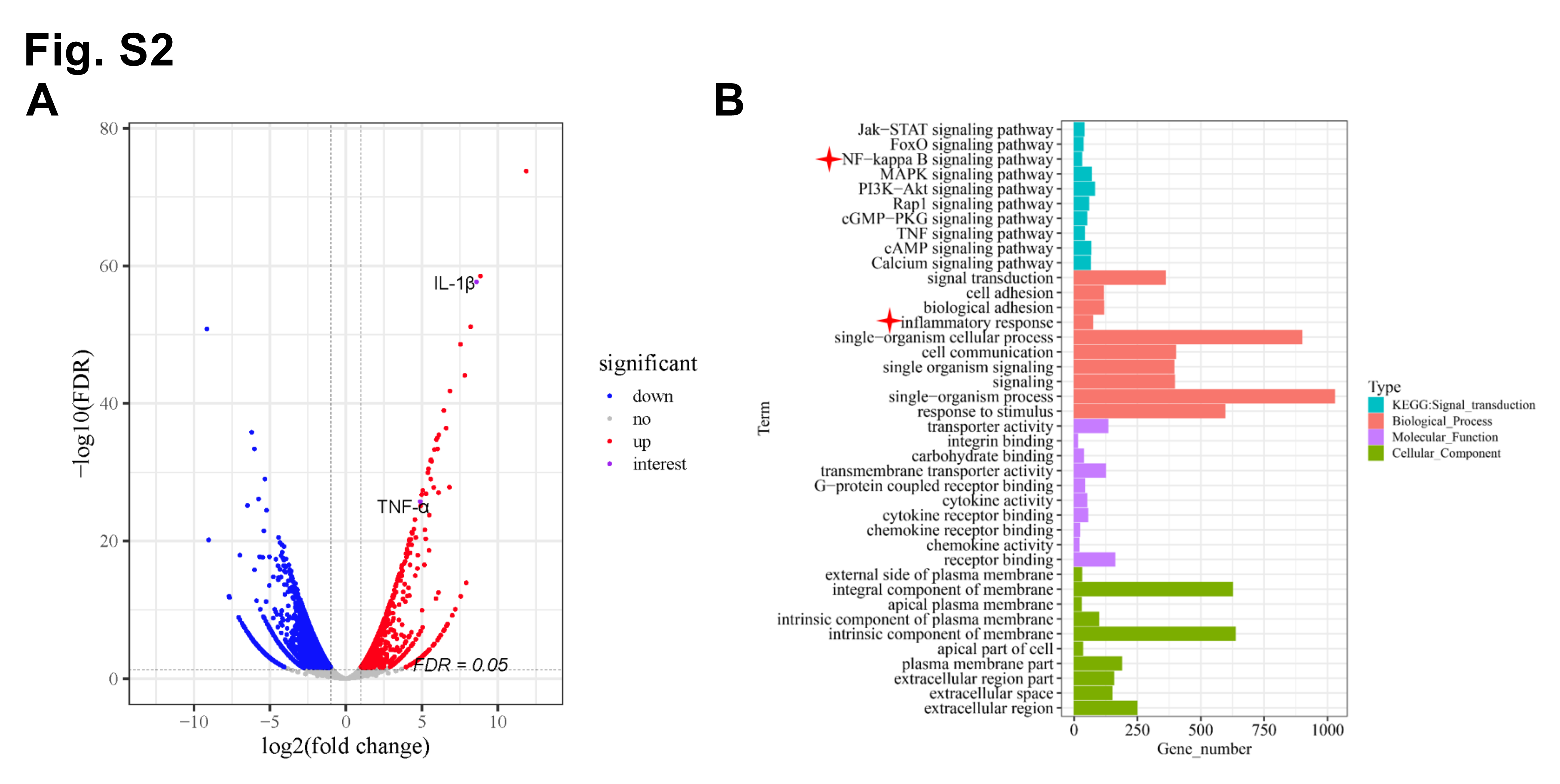

Supplement: Supplemental Material [file TEMI_A_2333381_SM3337.zip › Supplementary_Figures/FigS2.tif]

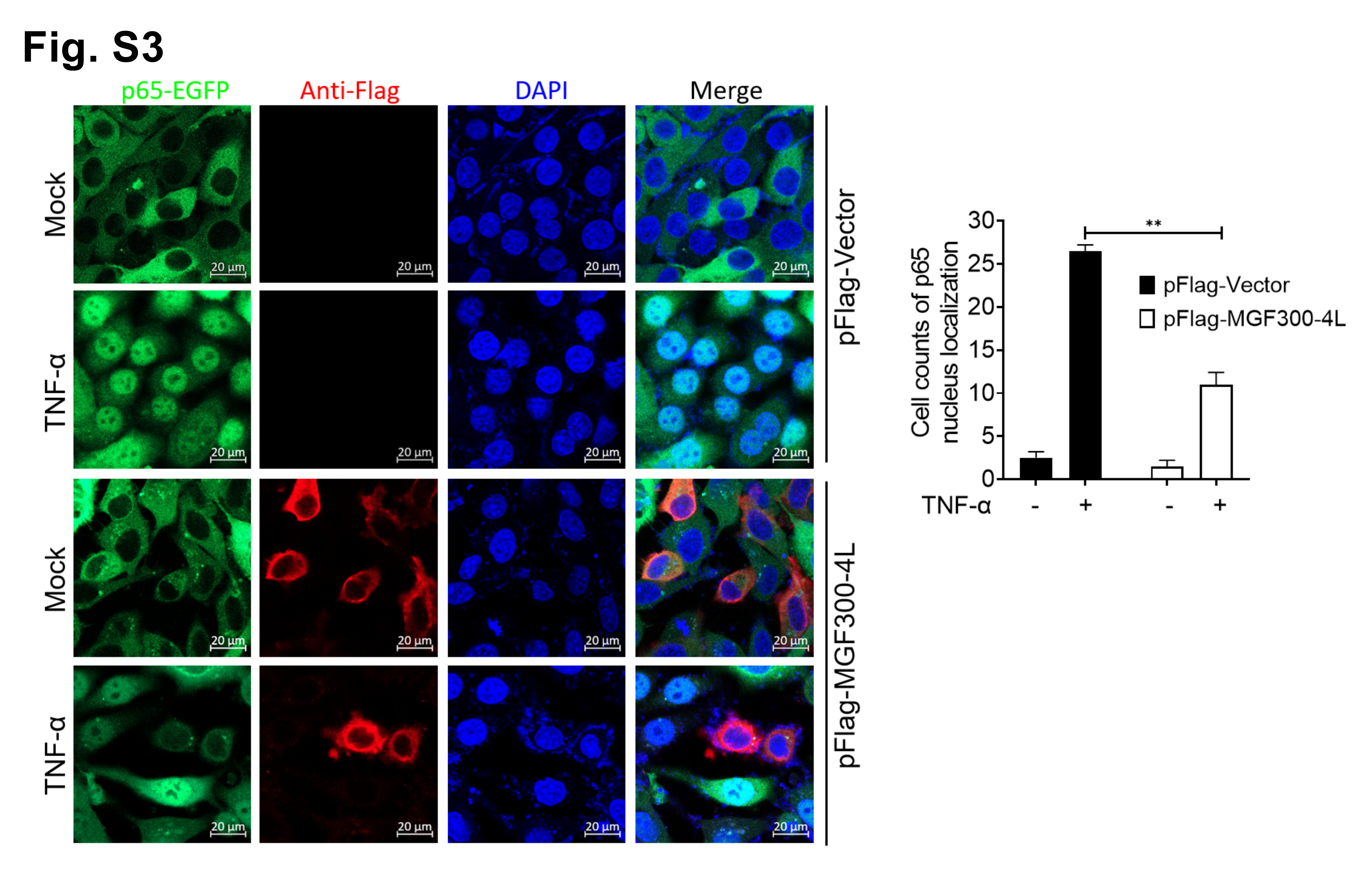

Supplement: Supplemental Material [file TEMI_A_2333381_SM3337.zip › Supplementary_Figures/FigS3.tif]

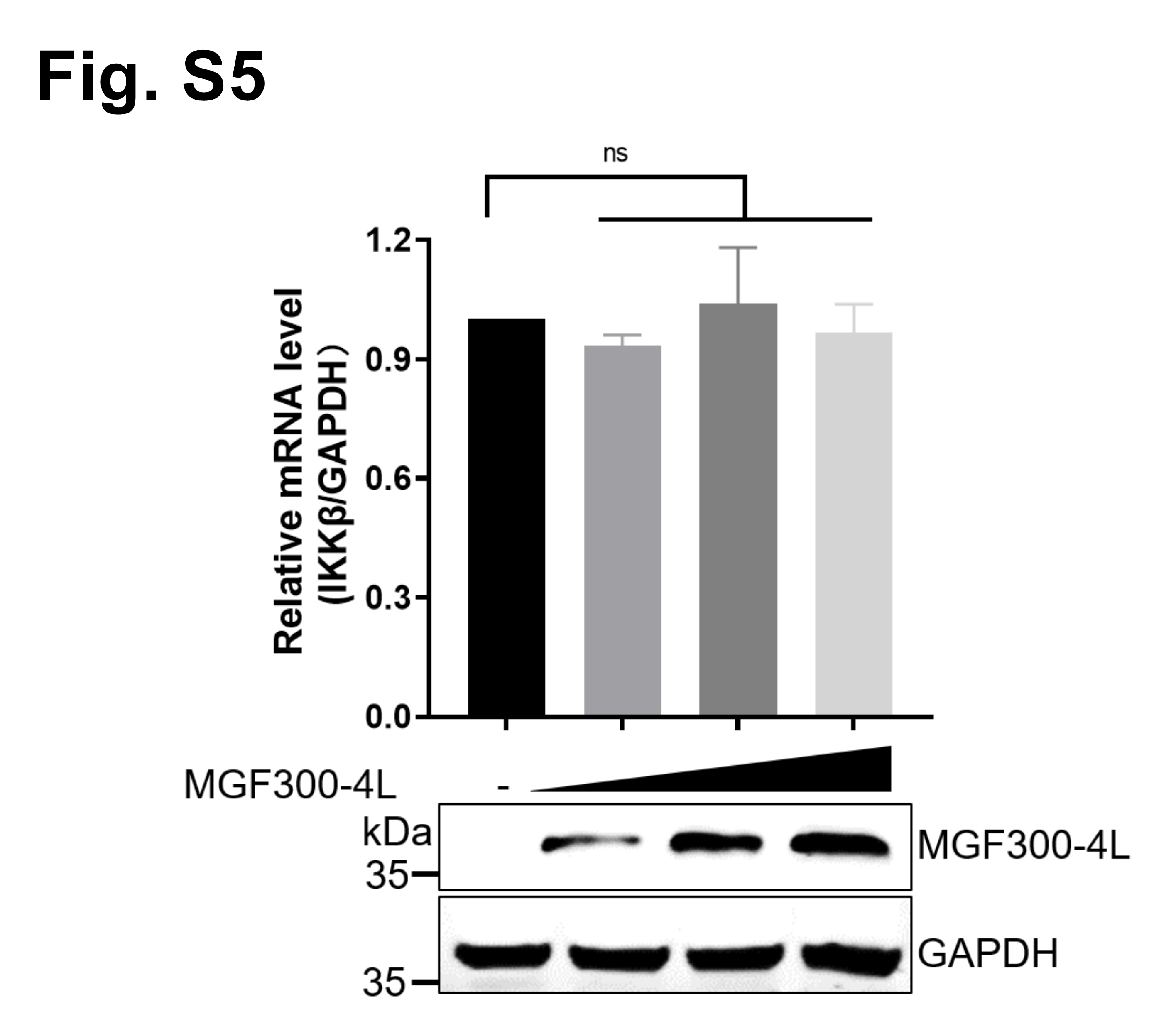

Supplement: Supplemental Material [file TEMI_A_2333381_SM3337.zip › Supplementary_Figures/FigS5.tif]

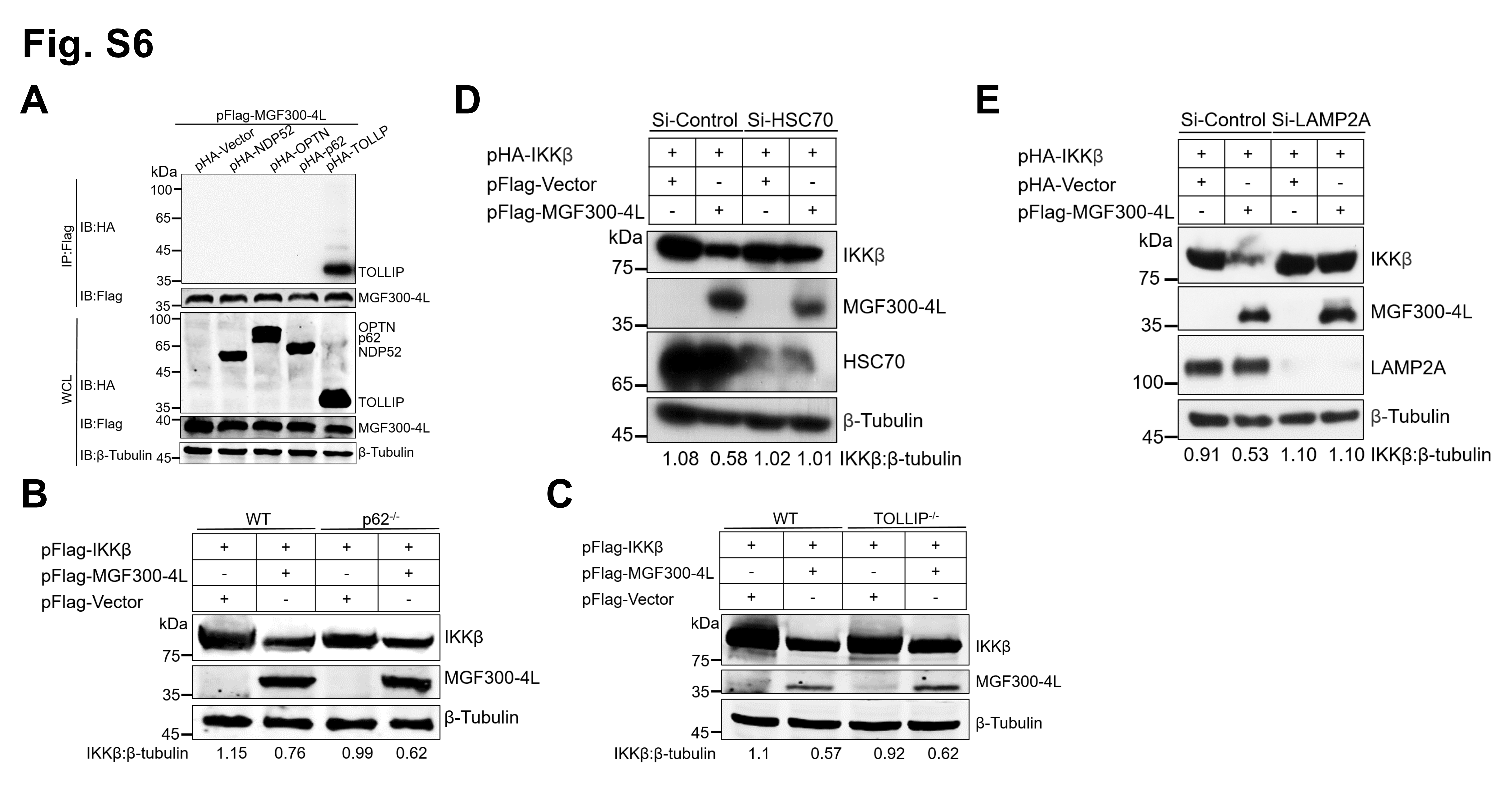

Supplement: Supplemental Material [file TEMI_A_2333381_SM3337.zip › Supplementary_Figures/FigS6.tif]

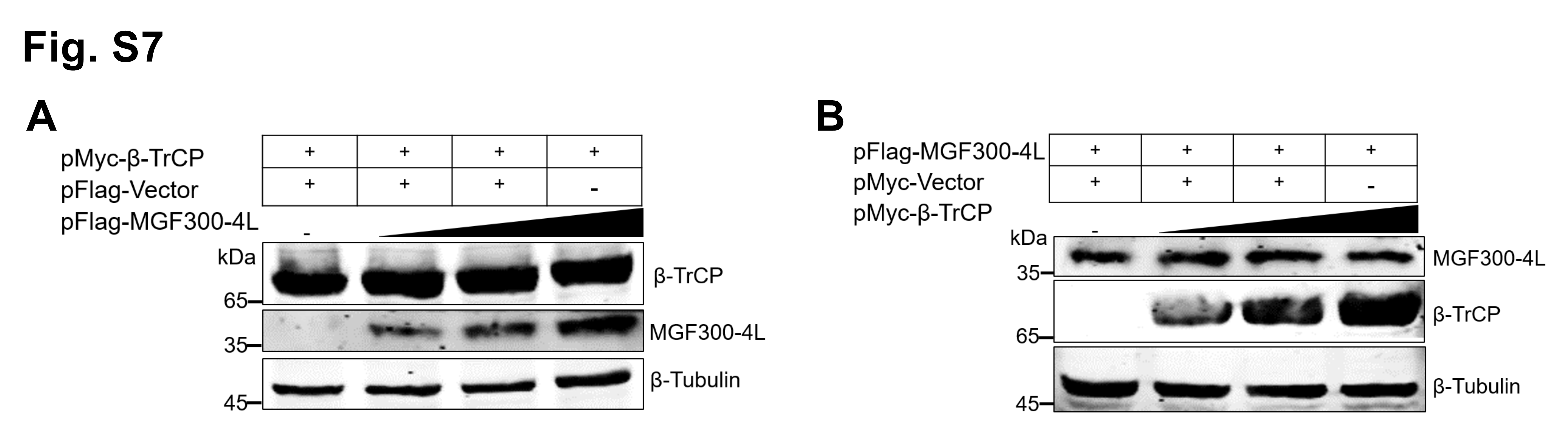

Supplement: Supplemental Material [file TEMI_A_2333381_SM3337.zip › Supplementary_Figures/FigS7.tif]

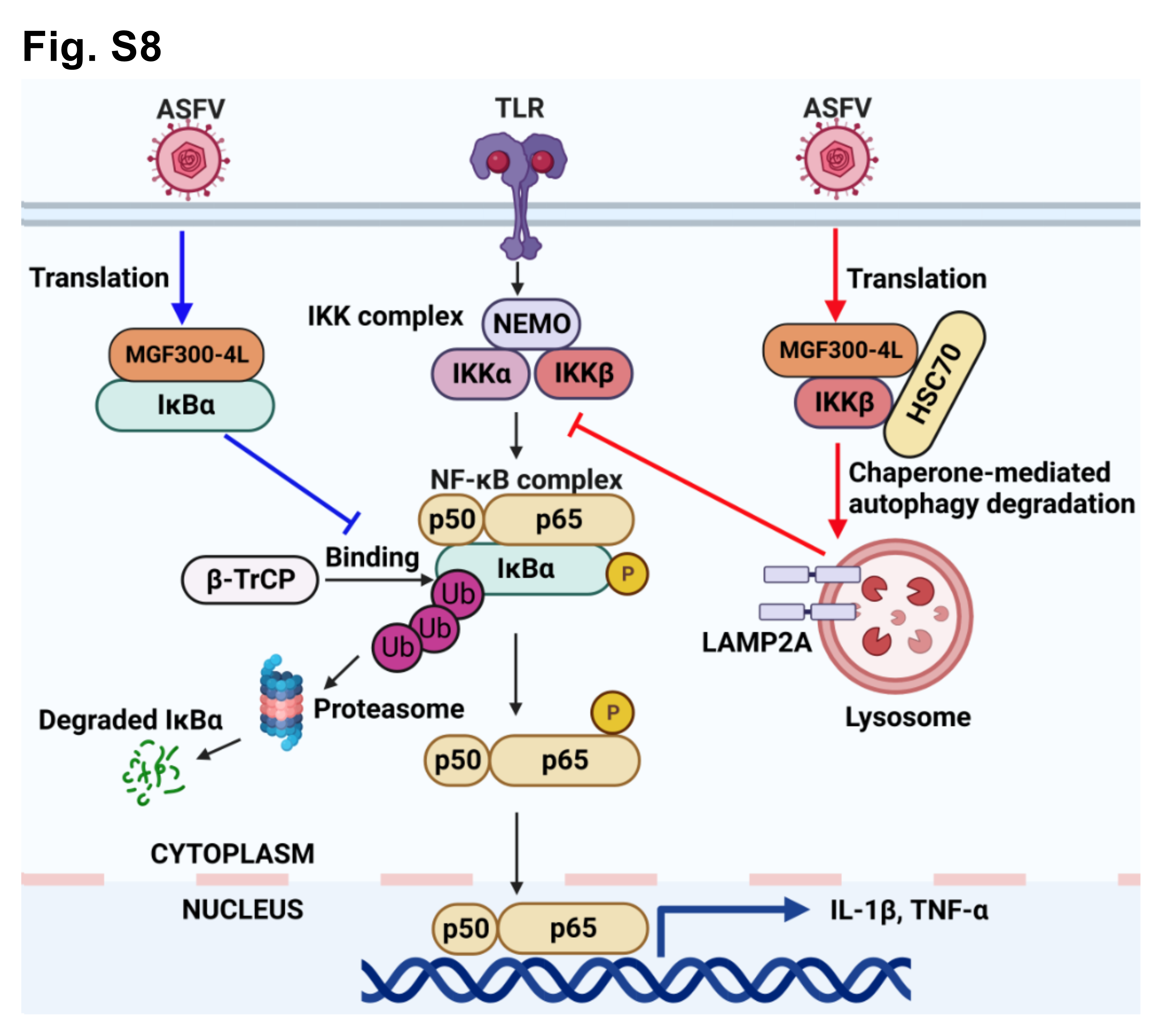

Supplement: Supplemental Material [file TEMI_A_2333381_SM3337.zip › Supplementary_Figures/FigS8.tif]

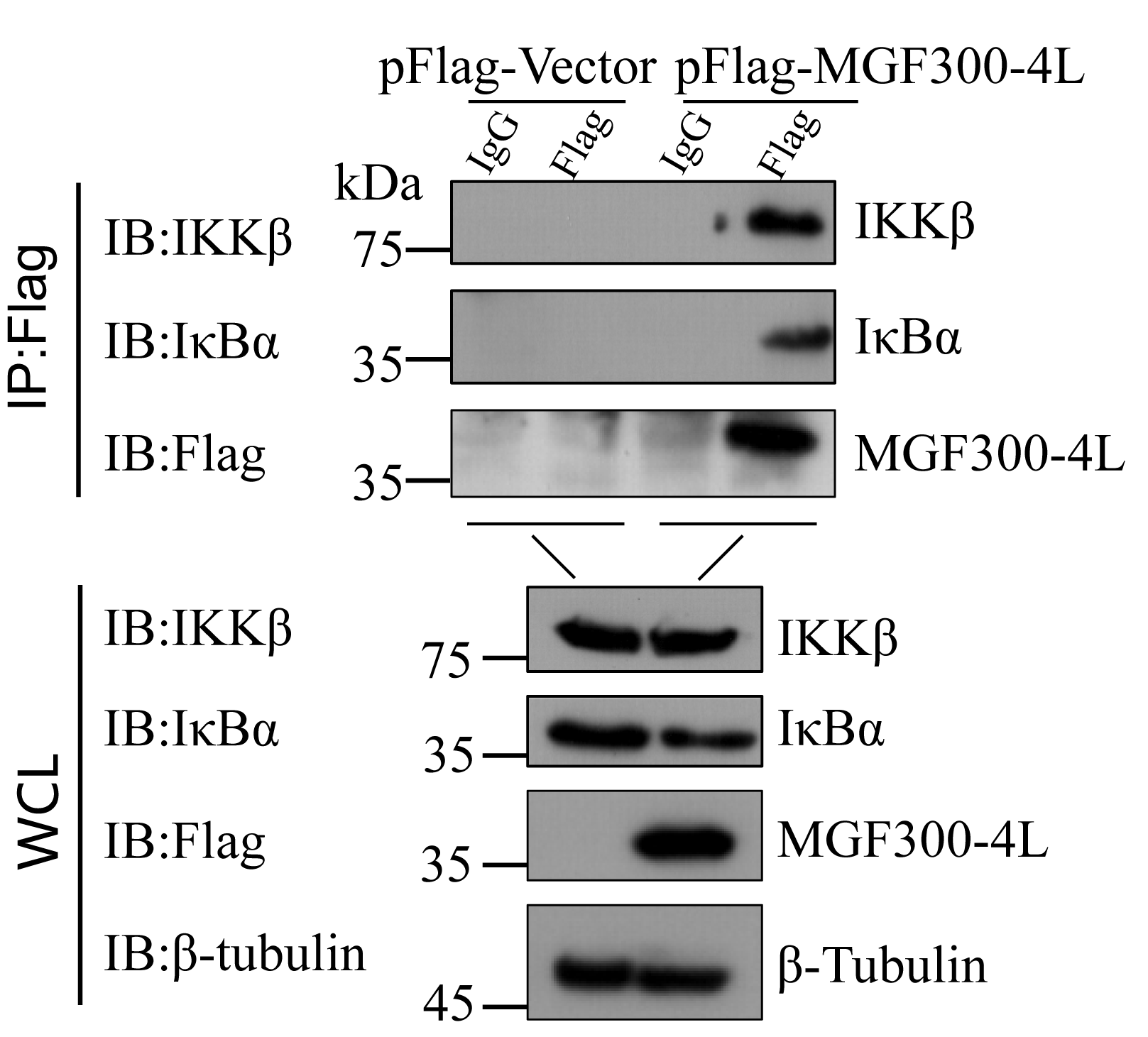

Supplement: Supplemental Material [file TEMI_A_2333381_SM3337.zip › Supplementary_Figures/Updated_Figure_S4.tif]
