# Supplementary material for: The African swine fever virus MGF300-4L protein is associated with viral pathogenicity by promoting the autophagic degradation of IKKβ and increasing the stability of IκBα
Source: Emerg Microbes Infect. 2024 Mar 19;13(1):2333381. doi: 10.1080/22221751.2024.2333381 (PMC11018083; doi:10.1080/22221751.2024.2333381)
Supplement: Supporting_information_updated_V2_1 [file TEMI_A_2333381_SM3235.docx]

**Supporting information**

**Figure S1. Characterization of the ASFV MGF300-4L protein.** (A) Conservation of the MGF300-4L protein in genotypes I and II ASFV strains. The MGF300-4L protein from ten ASFV strains was selected from the GenBank database to conduct a multiple sequence alignment using the Clustal W algorithm. The amino acid identities of MGF300-4L between various strains are 92% to 100%. (B) Subcellular localization of MGF300-4L. HEK293T cells were transfected with pFlag-MGF300-4L or pFlag-Vector. At 24 hours posttransfection, the subcellular localization of MGF300-4L in the HEK293T cells was examined by laser confocal microscopy. Scale bar, 20 *μ*m. (C) The MGF300-4L protein expression kinetics. PAMs were infected with the ASFV HLJ/18 strain (MOI = 5). At 2, 6, 8, 12, 15, 24, and 24 hours postinfection, the transcriptional levels of the *MGF300-4L*, *p72*, and *p30* genes were determined by RT-qPCR.

**Figure S2. Gene expression profiling in the Del4L-infected PAMs by RNA-seq analysis.** PAMs were either mock-infected or infected with Del4L or ASFV-WT (MOI = 5). A volcano plot was used to depict the gene expression changes in the Del4L-infected vs the ASFV-WT-infected PAMs (A). Upregulated and downregulated differentially expressed genes (DEGs) were represented by red and blue dots, respectively. Upregulated DEGs were compared between the Del4L and ASFV-WT groups by gene ontology (GO) category functional enrichment analysis. The analysis included three categories: biological process, molecular function, and cell component. Additionally, Kyoto Encyclopedia of Genes and Genomes (KEGG) pathway analysis was performed. The red asterisks indicate the signaling pathways of interest (B).

**Figure S3. MGF300-4L inhibits the TNF-*α*-triggered nuclear translocation of p65.** The HeLa cells stably expressing p65-EGFP were transfected with pFlag-Vector or pFlag-MGF300-4L. At 24 hours posttransfection (hpt), the cells were treated with or without TNF-*α* (10 ng/mL) for 5 min and then subjected to laser confocal microscopy. The cells (*n* = 30) expressing nuclear p65 in each group were counted. * *P* < 0.05; ** *P* < 0.01; *** *P* < 0.001; and ns, not significant (*P* > 0.05).

**Figure** **S4. The MGF300-4L protein interacts with IKK*β* and I*κ*B*α*.** HEK293T cells were transfected with pFlag-Vector or pFlag-MGF300-4L. At 24 hours posttransfection, the cells were lysed and immunoprecipitated by anti-Flag or IgG antibodies. The whole cell lysates (WCL) and immunoprecipitates were analyzed by immunoblotting with the indicated antibodies.

**Figure** **S5. The effects of MGF300-4L on the expression of IKK*β*.** HEK293T cells were transfected with an increasing amount of pFlag-MGF300-4L (0.5, 1, and 1.5 *μ*g). At 24 hours posttransfection, the mRNA level of IKK*β* was quantified by RT-qPCR. The protein level of MGF300-4L was examined by immunoblotting.

**Figure** **S6.** **MGF300-4L facilitates the degradation of IKK*β* independently of macroautophagy.** (A) The MGF300-4L protein interacts with TOLLIP. HEK293T cells were cotransfected with pFlag-MGF300-4L and the indicated Flag-tagged cargo receptors. At 24 hours posttransfection (hpt), the cells were lysed and the whole cell lysates (WCL) and immunoprecipitates were analyzed by immunoblotting (IB) using the indicated antibodies. (B and C) MGF300-4L degrades IKK*β* independently of p62 or TOLLIP. pFlag-IKK*β* and pFlag-MGF300-4L were cotransfected into the *p62*-knockout (*p62^-/-^*) (B), or *TOLLIP^-/-^* (C), or wild-type (WT) HEK293T cells. At 36 hpt, the expression levels of IKK*β* and MGF300-4L were analyzed by IB. (D and E) MGF300-4L degrades IKK*β* via CMA. HEK293T cells were cotransfected with pFlag-MGF300-4L, pHA-IKK*β*, and scramble or HCS70- (D) or LAMP2A-specific (E) siRNAs. The protein expression levels of IKK*β*, MGF300-4L, HSC70, and LAMP2A were analyzed by IB at 24 hpt.

**Figure** **S7. MGF300-4L interacts with *β*-TrCP but does not affect the stability of *β*-TrCP.** (A) Exogenous expression of MGF300-4L does not affect the expression of *β*-TrCP. HEK293T cells were cotransfected with pMyc-*β*-TrCP or pFlag-Vector and an increasing amount of pFlag-MGF300-4L (0.5, 1, and 1.5 *μ*g). At 24 hours posttransfection (hpt), the cell lysates were then analyzed by immunoblotting (IB). (B) Exogenous expression of *β*-TrCP does not affect the expression of MGF300-4L. HEK293T cells were cotransfected with pFlag-MGF300-4L and an increasing amount of pMyc-*β*-TrCP (0.5, 1, and 1.5 *μ*g). At 24 hpt, the cell lysates were analyzed by IB.

**Figure S8. Schematic diagram of the NF-*κ*B signaling pathway being inhibited by the ASFV MGF300-4L protein.** The MGF300-4L protein is expressed upon ASFV infection. The MGF300-4L protein interacts with both IKK*β* and I*κ*B*α*, which promotes the chaperone-mediated autophagic degradation of IKK*β* (red arrows) and stabilizes I*κ*B*α* (blue arrows), serving as a suppressor that prevents the activation of the NF-*κ*B signaling pathway (black arrows). Created with BioRender (https://biorender.com).

**Table S1. The primers used in this study.**
